# Selective ionization of oxidized lipid species using different solvent additives in flow injection mass spectrometry

**DOI:** 10.1007/s00216-023-04988-x

**Published:** 2023-10-17

**Authors:** Eleni Lazaridi, Marie Hennebelle, Boudewijn Hollebrands, Jos Hageman, Jean-Paul Vincken, Hans-Gerd Janssen

**Affiliations:** 1 Laboratory of Food Chemistry, Wageningen University & Research, Wageningen, the Netherlands; 2 Laboratory of Organic Chemistry, Wageningen University & Research, Wageningen, the Netherlands; 3 Unilever Food Innovation Center, Wageningen, the Netherlands; 4 Biometris, Applied Statistics, Wageningen University & Research, Wageningen, the Netherlands

**Keywords:** Lipid oxidation, Oxidized triacylglycerols, Triacylglycerols, Mass spectrometry, Flow injection

## Abstract

Lipid oxidation in food products is a crucial problem that causes undesirable changes in the food’s flavor, texture, and nutritional value. It should be carefully monitored as it can lead to the formation of potentially toxic compounds and in that way reduce the shelf life of the product. Liquid chromatography coupled to mass spectrometry is a powerful tool to monitor the formation of oxidized lipids. However, the presence of lipid species in both their non-oxidized and oxidized forms at distinctly different concentrations can hinder the detection and identification of the less abundant oxidized species, due to coelution. In this study, a flow injection mass spectrometry approach was used to selectively ionize oxidized triacylglycerols versus their non-oxidized precursors. Three mobile phase additives were investigated (ammonium formate, sodium acetate, and sodium iodide) at three different concentrations, and ion source settings (i.e., sheath gas temperature, capillary voltage, and nozzle voltage) were optimized. A fractional factorial design was conducted to examine not only the direct effect of the operating parameters on the selectivity of ionization for the oxidized lipid species, but also to assess their combined effect. Overall, selective ionization of oxidized versus non-oxidized lipid species was favored by the use of sodium-containing solvent additives. The application of specific ion source settings resulted in an increased ionization selectivity, with sheath gas temperature and capillary voltage having the most significant influence. A selectivity factor as high as 120 could be reached by combining 0.1 mg/mL sodium-containing additives, with 250 °C sheath gas temperature and 5000 V capillary voltage. These findings will contribute to future studies on fast detection and relative quantification of low abundant oxidized triacylglycerols and their possible impact on human health.

**Graphical abstract:**

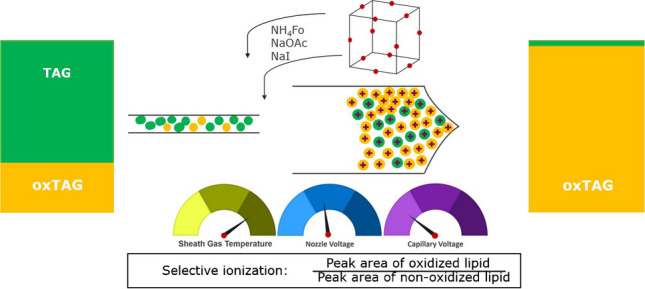

**Supplementary information:**

The online version contains supplementary material available at 10.1007/s00216-023-04988-x.

## Introduction

Lipid oxidation causes quality deterioration in lipid-rich food products leading to the formation of off-flavors and reduced shelf lives. Eventually, it can have adverse effects not only on food quality, but also on human health. For instance, lipid oxidation products have been associated with cardiovascular diseases [1], but can also cause an inflammatory response in organs such as liver, kidney, lung, and the gut itself [2], albeit that their true effects are largely misunderstood. This is at least partly because of difficulties in the analysis of trace levels of lipid oxidation species in common diets. The classical, wet-chemical analytical methods currently used to assess lipid oxidation in food products (e.g., peroxide and *para*-anisidine value, TBARs, hexanal) focus on one (type of) oxidation product and fail to grasp the chemical and multiscale complexity of lipid oxidation reactions. To ensure food safety, more detailed characterization of various oxidation intermediates and products is essential. The main analytical method used to characterize oxidized lipid samples is liquid chromatography-mass spectrometry (LC–MS). However, in complex food systems, the joint presence of lipid species in both their non-oxidized and oxidized forms at distinctly different concentrations (i.e., from 10 to 1000 times or higher excess of the non-oxidized species compared to oxidized ones) leads to limited or no detection of the less abundant species due to either overlap with the main compounds (i.e., co-elution), competition for ionization (i.e., ion suppression), or concentrations below the limit of detection. Clearly, novel analytical tools are needed that allow the characterization of the highly complex mixtures of oxidized species encountered in aged food systems.

Mass spectrometry is an indispensable technique in the analysis of oxidized lipids. It is used either in LC–MS-based methods [3, 4] or in direct inlet or flow injection based strategies [4–6]. For LC–MS analysis, highly efficient LC set-ups or even multidimensional chromatography systems are typically used to provide detailed compositional information on both the oxidized and non-oxidized lipid species present, exploiting both retention and mass spectral information [7–10]. Unfortunately, these methods can be time consuming. Moreover, optimization of the ionization conditions is important to avoid ionization suppression and obtain a high sensitivity to allow analysis of the main and trace compounds in one run.

In contrast to LC–MS-based methods, direct inlet or flow injection methods are much faster. Moreover, the ionization efficiency of individual lipids does not vary due to variations in mobile phase composition, as would be the case in gradient LC–MS methods, facilitating method optimization. Finally, these methods can provide virtually unlimited acquisition time at a constant concentration, which allows to perform tandem mass spectrometric mapping with multiple fragmentation strategies [11]. However, due to lack of chromatographic separation, there are also some issues related to direct inlet or flow injection–based methods that require consideration. The main ones are, as in LC–MS, the potential occurrence of ion suppression and the risk of false discoveries due to interferences from species having similar fragmentation pathways as the species of interest [12]. Another reported shortcoming of direct inlet or flow injection MS in complex mixtures is the occurrence of selective ionization, holding the risk that specific species of compounds remain undetected [13]. On the other hand, if ionization conditions can be tuned to provide a sufficiently high selectivity towards oxidized species, this would resolve the interference of non-oxidized species of similar masses, opening possibilities for rapid direct MS analysis of oxidized lipids in lipid extracts from partially oxidized foods.

Among the various types of ionization methods for comprehensive analysis of intact lipids, electrospray ionization (ESI) is the most widely used [14]. Due to the lack of acidic and basic groups, neutral lipids are typically detected by ESI–MS using addition of various salts to the solvent or eluent. Published research on the salt to be used, as well as its concentration and other ionization parameters, mostly focused on enhancing the overall sensitivity of the MS detection but did not address the possibility to obtain selective ionization of oxidized versus non-oxidized species. Interestingly, optimum conditions found are rather different for oxidized (ox) versus non-oxidized (non-ox) species. As an example, improved ionization of non-ox triacylglycerols (TAGs) in ESI–MS positive mode was obtained with the addition of ammonium formate (NH_4_Fo) [15, 16] or ammonium acetate (NH_4_OAc) [17, 18], whereas for the analysis of ox TAGs, sodium salts such as sodium acetate (NaOAc) and sodium iodide (NaI) were preferred [8, 19]. Since these previous studies focused either on non-ox or ox compounds, it is hard to say whether selective ionization can be obtained with specific solvent additives under conditions optimized for maximum selectivity rather than maximum sensitivity. Additional selectivity can also be obtained by focusing on selected masses of the oxidized and non-oxidized species. Exploiting the three options mentioned above, i.e., type of solvent additive, MS ionization settings, and monitoring of selected masses, will allow to introduce an increasing level of selectivity in the analysis of lower levels of oxidized species. Depending on the degree of selectivity that can be achieved, this would either enable the use of direct inlet MS for measuring oxidized species in edible oil, or it would at least reduce the requirements to be imposed on the chromatographic separations.

In this study, we investigated the strategy of exploiting selective ionization in the analysis of oxidized lipid species. Flow injection MS experiments were used to identify conditions for selectively ionizing and detecting oxidized lipid species while suppressing the signals of their non-oxidized precursors (i.e., TAGs). Different solvent additives were investigated at different concentrations. The impact of ion source settings, such as sheath gas temperature, nozzle, and capillary voltage, was also evaluated to obtain the highest possible sensitivity for oxidized species at the lowest possible sensitivity for their non-oxidized counterparts. A fractional factorial design (FrFD) was conducted for parameter optimization to examine not only the direct effect of the operating parameters on the selectivity of ionization of the oxidized lipid species, but also to assess their combined effect. Finally, the ability to obtain additional selectivity by evaluating the data focusing on either specific mass ranges reflecting specific groups of oxidized species, or on specific masses representing specific oxidized compounds, was evaluated.

## Materials and methods

### Chemicals and materials

#### Chemicals

Chloroform (CHCl_3_, stabilized with ethanol), methanol (MeOH, ULC/MS-CC/SFC grade), *n*-hexane, 2-propanol (IPA, ULC/MS-CC/SFC grade), and ammonium formate (NH_4_HCO_2_, ULC/MS-CC/SFC grade) were purchased from Biosolve (Valkenswaard, the Netherlands). Sodium acetate (NaO_2_C_2_H_3_) and sodium iodide (NaI, ACS reagent, ≥ 99.5%) were purchased from Sigma-Aldrich (Zwijndrecht, the Netherlands). LiChrosolv ethanol (EtOH, gradient for liquid chromatography) was purchased from Merck (Darmstadt, Germany).

#### Standards and oil

The TAGs triolein (C18:1/C18:1/C18:1) and 1,2-olein-3-linolein (C18:1/C18:1/C18:2) were purchased from Larodan (Solna, Sweden). Unilever Research (Wageningen, the Netherlands) provided a commercial fresh (non-oxidized) rapeseed oil. For method development, a highly oxidized rapeseed oil sample and oxidized TAG standards were produced using an accelerated aging protocol at 60 °C for 12 days as described in our previous work [10]. Stock solutions of each sample and standards were prepared by dilution in CHCl_3_/MeOH (2:1) to a concentration of 1 mg/mL. All experiments were conducted using a final concentration of 0.1 mg/mL in IPA for both types (oxidized and non-oxidized) of standards and oils. These samples were prepared by diluting the initial stock solution 10 times.

#### Estimation of oxidized lipid species by NMR

Semi-quantitative characterization of the oxidized standards and oil samples was conducted through the use of two complementary NMR methods. Hydroperoxides and aldehydes were quantified with a single pulse and band-selective ^1^H NMR method [20], while epoxides were quantified using a ^1^H-^13^C HSQC NMR method [21]. Estimating the concentrations of non-oxidized and oxidized lipid species in our samples was essential to normalize the MS signal (“Data processing” section) and calculate the selectivity factors.

The fresh oil contained less than 0.01 mol hydroperoxide groups per kg TAG and neither epoxides nor aldehydes. The oxidized rapeseed oil contained 0.022 mol aldehyde groups per kg TAG, 0.167 mol epoxide groups per kg TAG, and 0.236 mol hydroperoxide groups per kg TAG. An exact description of all (oxidized-)TAGs present is impossible, but by making a few assumptions, a reasonable impression of the oxidized oil can be obtained. Two assumptions were made. Firstly, we assumed that a fatty acid only contains either no, or just one oxidized group and we neglected the occurrence of fatty acids with more than one oxidized group, as these are highly unlikely to occur in the mono-unsaturated olein, the most abundant unsaturated fatty acid in our samples. In terms of oxygens, this means a fatty acid contains no added oxygen, while its oxidized products contain either one additional oxygen (aldehyde or epoxide) or two added oxygens (hydroperoxides). Next, we assumed an average molecular mass for the TAG molecules of 885 g/mol, i.e., the mass of triolein (OOO), the most abundant TAG in rapeseed oil. The latter means that 1 kg of oil translates to 1.13 mol of TAG and consequently, to 3.39 mol of fatty acids (FA). Since our oil contains 0.189 mol 1ox FA (epoxide or aldehyde)/3.39 mol FA, it means that there is 0.056 mol of 1ox FA per mol FA. Similarly, we have 0.07 mol 2ox FA (hydroperoxide) and 0.874 mol of non-ox FA per mol FA. The three different oxidation degrees of the FAs (0, 1, or 2ox) and three possible positions on the glycerol backbone (*sn* 1, 2, 3) resulted in 27 different TAG combinations, for which it was possible to estimate the chance of occurrence from the levels of the oxidized fatty acids determined using NMR (see Supplementary Material Table S2). Along these lines, the chances to have a TAG with no oxygen groups, one, two, three, or four oxygens (abbreviated as 1ox TAG, 2ox TAG, 3ox TAG, or 4ox TAG) in oxidized rapeseed oil were estimated to be 66.76%, 12.83%, 16.86%, 2.07%, and 1.35%, respectively. The chance of having a TAG with five or six oxygens (5 and 6ox TAG) was included in the calculations, but the occurrence of these species was neglected since they would account for less than 0.5%.

Similar calculations were applied to estimate the chance to have a TAG with no oxygen groups, 1ox TAG and up to 6ox TAG for the oxidized OOO and OOL standards (Supplementary Material Table S2).

### Instrumentation and conditions

#### Flow injection electrospray ionization mass spectrometry (ESI/MS)

Direct inlet mass spectrometry (MS) experiments were performed on an Agilent 6460 triple quadrupole mass spectrometer (Agilent, Amstelveen, the Netherlands). An Agilent 1200 series binary pump and autosampler were used to deliver the samples to the ion source. The eluent used was *n*-hexane/IPA (1:1) at a flow rate of 300 μL/min. The injection volume was 5 μL. The solvent additives were dissolved in EtOH/IPA (1:1) and delivered to the ion source using an external syringe pump equipped with a 10 mL Hamilton glass syringe, at a flow rate of 50 μL/min. The LC flow and additive flow from the external pump were combined in a T-piece prior to the MS. The mass spectrometer was operated in positive ESI mode (full scan) with the mass scan range set from *m*/*z* 100 to 1000. Ion source parameters sheath gas temperature, capillary voltage, and nozzle voltage were tested at three levels: high, medium, and low (Table 1). Drying gas flow rate was 7 L/min at 300 °C, nebulizer pressure was 45 psi, and a 11 L/min sheath gas flow was applied. All measurements were performed in duplicate. Acquisition of the MS data was performed using Agilent MassHunter 10.0 software.

**Table 1:** Selected conditions for optimization of direct infusion MS method

A	B	C	D	E
Type of solvent additive	Concentration of solvent additive (mM)	Sheath gas temperature (°C)	Capillary voltage (V)	Nozzle voltage (V)
Ammonium formate (NH_4_Fo)	5	150	2000	500
	10	250	3500	1000
	20	350	5000	1500
Sodium acetate (NaOAc)	0.05			1500
	0.1			
Sodium iodide (NaI)	0.2			

### Fractional factorial design

In this study, the effect of five different factors on the selectivity of ionization was investigated. The five factors studied were: (A) type of solvent additive, (B) concentration of the solvent additive, (C) sheath gas temperature, (D) capillary voltage, and (E) nozzle voltage. Each factor was studied at three different levels as described in Table 1. Conditions were selected to cover the entire allowed working range of the instrument including also the recommended default conditions. To limit the number of experiments, while maximizing the information obtained, a 3^5^ fractional factorial design (FrFD) was applied using the R package Planor [22]. The script of this model is provided in Supporting Material. This FrFD allowed us to estimate the main independent effects and two-way interactions that could affect the selective ionization of oxidized versus non-oxidized lipid species. A total of 81 combinations were investigated (Supporting Material Table S1).

### Data processing

In order to evaluate the selectivity of ionization of oxidized lipid species versus their non-oxidized precursors, the ratio of normalized peak areas of the oxidized lipids over that of the non-oxidized form was calculated. Normalization was performed by dividing absolute peak areas by the concentration of the group in the standard or sample as determined by NMR (see “Estimation of oxidized lipid species by NMR” section), according to Eq. 1.

1 $$\mathrm{Selectivity\;}=\;\frac{Peak\;area\;oxTAG\;/\;NMR\%\;oxTAG}{Peak\;are\;nonoxTAG\;/ NMR\%\;nonoxTAG}$$

**Fig. 1:**
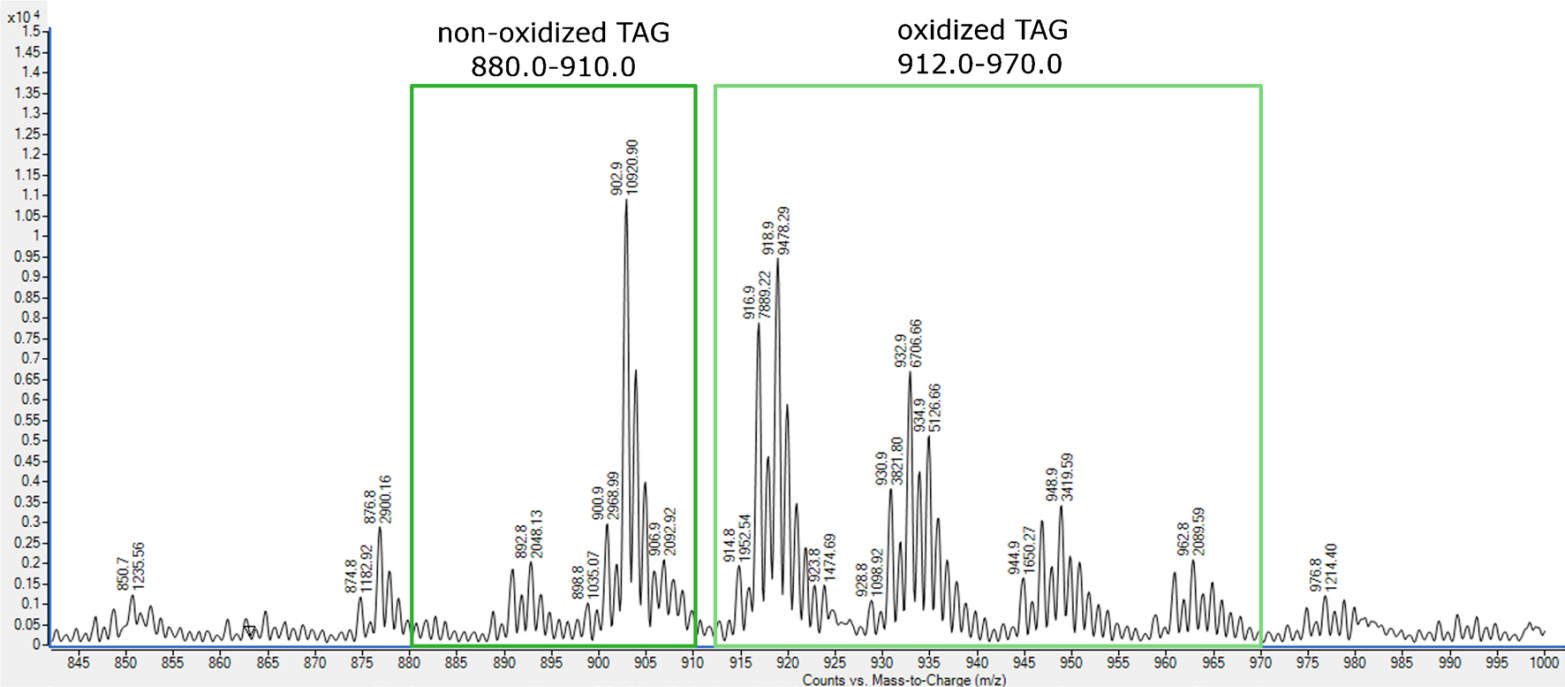
Flow injection mass spectrometric analysis of oxidized rapeseed oil indicating *m*/*z* regions selected for the first stage of selectivity. Data were acquired under condition set #15 (0.1 mM NH_4_Fo, 250 °C sheath gas temperature, 5000 V capillary voltage, and 1000 V nozzle voltage)

The first stage in our approach was based on monitoring mass ranges. It exploited two aspects of selectivity: the ionization selectivity, i.e., differences in ionization between oxidized and non-oxidized species upon ionization, and the mass range selectivity, i.e., differences in *m*/*z* induced by the addition of one or more oxygens in the oxidized lipid species (oxTAG) as compared to their non-oxidized forms (TAG). This approach aims to provide a first overall estimation of the selectivity obtained for oxidized versus non-oxidized species. The *m*/*z* regions from 880.0 to 910.0 Da and from 912.0 to 970.0 Da were selected for the non-oxidized and oxidized TAGs in the rapeseed oil sample, respectively. Figure 1 shows the mass spectrum obtained for the oxidized rapeseed oil at ionization condition set #15 (see Supporting Material Table S1). Clear regions of oxidized species with increasing numbers of oxygen groups can be seen for 1ox (m/z 914.0–926.0), 2ox (m/z 928.0–942.0), 3ox (m/z 944.0–957.0), and 4ox TAG (m/z 959.0–970.0). For the OOO TAG standard, the selected *m*/*z* regions for the non-oxidized species and the oxidized compounds were 884.0 to 908.0 Da and 915.0 to 970.0 Da, respectively. For the OOL TAG standard, the selected *m*/*z* regions for the non-oxidized species and the oxidized compounds were 883.0 to 906.0 Da and 913.0 to 968.0 Da. These mass ranges were selected to cover the majority of TAG species and adduct ions present in our samples, while minimizing overlap between the non-oxidized species of higher *m*/*z* and the mono-oxidized lightest species with lower *m*/*z*.

Since in this first stage of investigation some overlap is likely to be present (i.e., some low mass oxidized species might end up in the non-oxidized region and vice versa), a second stage was performed. In the second stage of investigation, we focused on specific, selected ions of specific oxidized and non-oxidized analytes. The selection of the ions of interest was based on the composition of the oil used. Rapeseed oil consists of two main TAGs, dioleoyl-linoleoyl-glycerol (OOL) and triolein (OOO) [23], and the most abundantly formed oxidized products were the ones containing one oxygen [1ox-TAG (TAG + 16)], two oxygens [2ox-TAG (TAG + 32)], and three oxygens [3ox-TAG(TAG + 48)]. In this stage of investigation, the different types of adducts formed for each of the aforementioned analytes of interest were also considered.

### Statistical data analysis

The significance of the individual factors and their two-way interactions were investigated quantitatively using analysis of variance (ANOVA). The quantitative dependent variable required for the ANOVA was the selective ionization factor. Tukey’s comparisons (with a significance level of *α* = 0.05) were performed to gain a better understanding of the effect of the various levels of the factors. All statistical analyses were performed on the second stage of the analysis (use of specific masses). ANOVA was performed using the R statistical software package [24]. The script used for the statistical analysis is provided in Supporting Material.

## Results and discussion

The detection of low amounts of oxidized lipid species in a sample with very high amounts of their non-oxidized precursor species requires a method that is both very sensitive and very selective. During this study, we examined the possibility of using flow injection MS analyses to selectively detect only the oxidized lipid species. As outlined in the “Introduction,” there are three options to improve selectivity of oxidized over non-oxidize species. Firstly, mobile phase additives and ionization settings can be selected such that the oxidized lipids will preferentially ionize (options 1 and 2) and next, specific mass ranges or specific masses, selective for the oxidized species, can be monitored (option 3). The resulting selectivity is the combined effect of these two steps, one being more ‘chemical’ in nature, i.e., selective ionization, the second more related to mass spectral properties. In practice, unfortunately, their individual contributions to the ionization selectivity cannot be assessed separately as a mass spectral read-out will always be needed. In our studies, we monitored two stages of selectivity. In the first stage, we looked at specific mass windows for the lighter non-oxidized species versus the heavier oxidized species; in the second stage, we narrowed the mass information by monitoring specific masses rather than mass windows, again with varying ionization conditions.

### First stage of selectivity: selective mass range

**Fig. 2:**
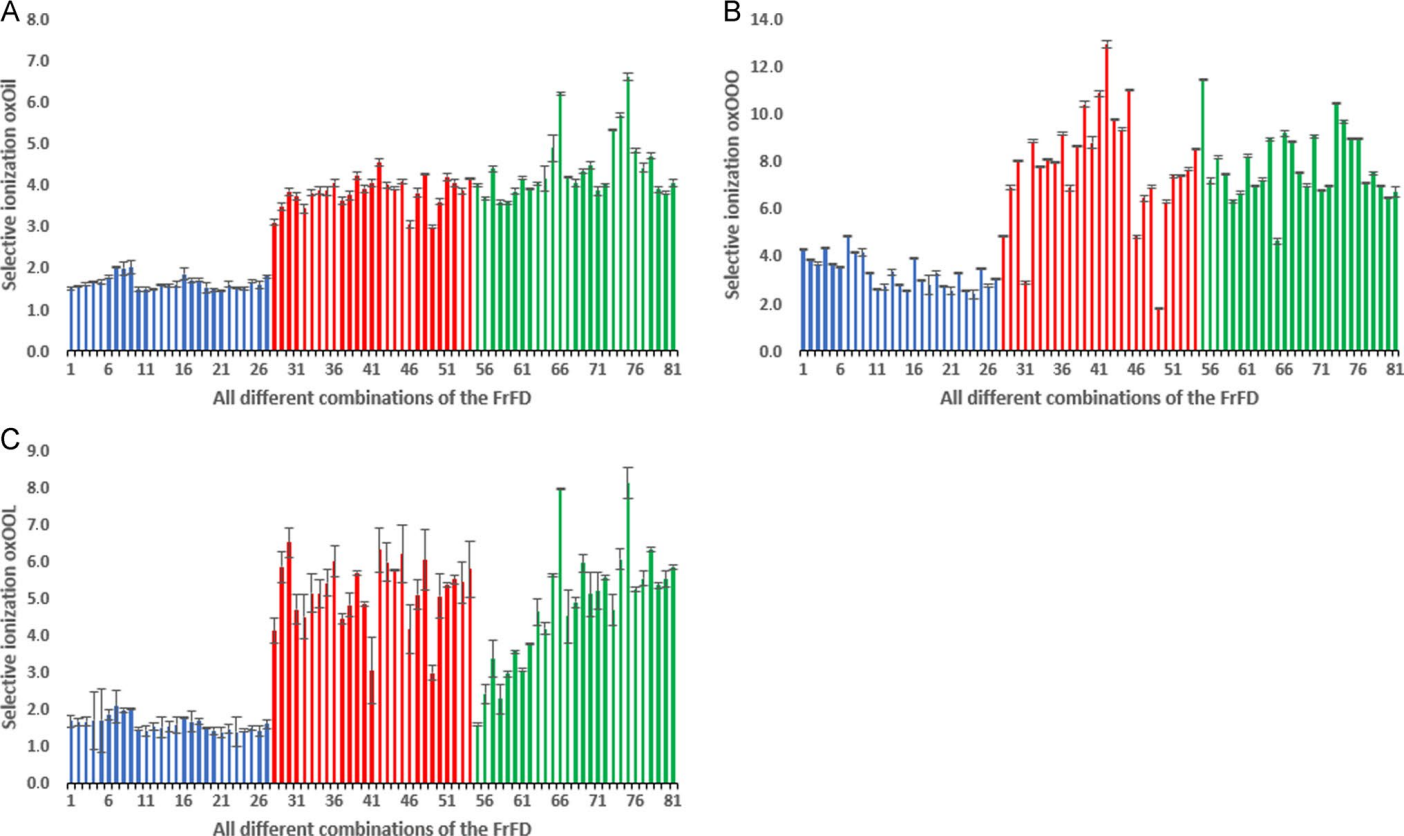
Selectivity factors of oxidized versus non-oxidized species for **A** oxidized rapeseed oil, **B** oxidized OOO standard, and **C** oxidized OOL standard. Subsequent bars indicate the results for the 81 conditions tested (details in Supplementary Material Table 1). In all bar plots, the blue, red, and green colored bars represent the experiments conducted using ammonium formate (NH_4_Fo), sodium acetate (NaOAc), and sodium iodide (NaI), respectively. Data shown are the averaged measurements ± SD

The selectivity results obtained for the oxidized oil and the oxidized TAG standards (OOO and OOL) are presented in Fig. 2A–C, respectively. The figures show the quantitative selectivity data for all 81 ionization conditions (Supporting Material Table S1) tested. An infinite selectivity is only obtained if (i) the non-oxidized species yield no signal and (ii) no fragmentation of oxidized species into the non-ox mass range occurs. The full set of quantitative data is given in the Supplementary Material Table S3. Selectivity values were obtained based on mass windows of oxidized and non-oxidized compounds and normalized using Eq. 1 (see “Data processing” section).

Concerning the oxidized rapeseed oil (Fig. 2A), an ionization selectivity of up to 6.6 was obtained when using 0.2 mM NaI, at 150 °C sheath gas temperature, 5000 V capillary voltage, and 500 V nozzle voltage (condition #75). This value means that if a sample would contain equal levels of oxidized and non-oxidized TAGs, the MS area of the window representing the oxidized species would be 6.6 times larger than that for the non-oxidized sample. For this sample, the highest selectivity was obtained when using sodium-containing solvent additives i.e., NaOAc (up to 4.5; red bars) and NaI (up to 6.6; green bars), whereas the least promising results were found with NH_4_Fo (selectivity only up to 2.0; blue bars). Our finding of sodium being the preferred additive is in line with findings from other authors that sodium-containing additives are preferred for the analysis of oxidized compounds [8, 19], whereas ammonium [15–18] presents a higher sensitivity for non-oxidized species. From the data presented in Fig. 2A, it is clear that the type of solvent additive had the most pronounced effect on the selectivity. The other factors (concentration, sheath gas temperature, and nozzle voltage) had only a modest influence. The only exception was the capillary voltage; when set at the highest tested level (5000 V), a slight increase (up to around two-fold) in the ionization selectivity was indeed observed (conditions # 3, 6, 9, 12, 15, 18, 21, 24). A detailed overview of the experimental conditions tested and the results found is given in Tables S1 and S3, respectively.

For real oil samples as the oxidized rapeseed oil shown in Fig. 2A, the selectivity that can be obtained from the use of separate mass windows for oxidized and non-oxidized species is slightly biased as a result of the overlapping mass ranges. This limitation is bidirectional in the sense that there can be both heavier non-oxidized species that fall in the mass window of the oxidized species and oxidized lighter species or fragments thereof that fall in the window of the non-oxidized species. An example of the latter case would be the so-called 2.5 glycerides, degradation products of hydroperoxy-TAGs consisting of two intact fatty acid chains and one short chain, often with an aldehyde functionality, resulting from the loss of a volatile secondary oxidation product [17]. These aldehyde-type oxidation products can have masses in the non-ox window. NMR results fortunately indicate aldehyde levels to be low (0.022 mol/kg at a total of 0.425 mol oxidized groups/kg); hence, the error made as a result of the 2.5 glycerides is probably small. On the other hand, also other oxidized species that fragment upon ionization might end up in the non-ox window, in that way resulting in lower overall selectivity values.

Performing similar studies with pure TAG standards in parallel with the study of real oils could potentially shed light on the relative importance of the contribution of ionization differences on the one hand, and mass-range selectivity on the other, to the overall selectivity factors found for the real oil sample. In doing so, however, it is important to stress that also with oxidized pure TAG standard errors can be made, albeit that selectivity limitations now are no longer bidirectional. For model triglycerides, in-source fragmentation of oxidized species could still cause their fragments to fall in the non-ox window. On the other hand, however, non-ox species cannot enter the mass window of the oxidized species. Figure 2B and C depict the results of the oxidized TAG standards OOO and OOL, respectively. The highest and lowest selectivity values differ by almost a factor 7 (condition sets #42 vs. #49, Supplementary Material Fig. S1). Again, the highest selectivities were found with sodium as the additive, albeit that the influence of the experimental settings seems a bit stronger. Selectivities as high as 13.0 were obtained for the oxidized OOO standard (Fig. 2B) when using 0.1 mM NaOAc, at 250 °C sheath gas temperature, 5000 V capillary voltage, and 1500 V nozzle voltage (condition #42). This means that under these conditions, equal masses of oxidized and non-oxidized OOO would result in a peak area almost 13 times higher for the oxidized species. As expected, the oxidized rapeseed oil more closely resembles the behavior of the OOL standard. Thermal oxidation of a fatty acid chain generally starts with the abstraction of a hydrogen atom from the bis-allylic site, which only the linoleic acid possesses. The rate of oxidation of polyunsaturated TAGs like OOL is hence higher than the one of monounsaturated TAGs such as OOO, leading to a higher amount of oxidation products from polyunsaturated TAGs. The rapeseed oil studied here is therefore expected to follow a trend more comparable to the oxidation of the OOL standard. Unfortunately, also the results with the pure standards do not allow us to draw sound conclusions regarding the relative importance of selective ionization vs. mass window selectivity. Products of oxidation of OOO are believed to be more stable resulting in less in-source fragmentation and hence no, or at least less, overestimation of the non-ox mass range is expected. If we assume this data bias to be negligible, oxidized OOO species would ionize up to 13 times better than non-oxidized OOO. However, since this factor is strongly TAG dependent, attributing the difference in the oxidized oil and standards to either selective ionization or mass range selectivity is not possible. To which extent focusing on specific masses for oxidized vs. non-oxidized lipids can resolve this issue is studied in the “Second stage of investigation: selected masses” section.

### Second stage of investigation: selected masses

A further improvement in selectivity can be obtained by monitoring specific masses instead of specific mass windows for the oxidized vs. non-oxidized triglycerides. This can be done either with extracted ion current mode (EIC) or using selected ion monitoring (SIM). These routes, although different in terms of sensitivity, in principle will not differ in terms of selectivity as that is a ratio of sensitivities. Here, EIC was used to allow posterior spectral evaluation when needed. Because single masses are selected, the target TAGs as well as the solvent additive chosen have to be selected prior to data interpretation. In this second stage of investigation, we focused on specific ions of oxidized and non-oxidized OOO and OOL, the two most abundant TAGs of rapeseed oil (Table 2).

**Table 2:** Ammoniated and sodiated adduct ions monitored during the investigation for OOO and OOL for oxidized standards and oil

		Non-ox	1ox	2ox	3ox
[M + NH_4_]^+^	OOO	902.2	918.2	934.2	950.2
	OOL	900.2	916.2	932.2	948.2
[M + Na]^+^	OOO	907.5	923.4	939.4	955.2
	OOL	905.5	921.2	937.2	953.2

#### Ammonium formate as solvent additive

**Fig. 3:**
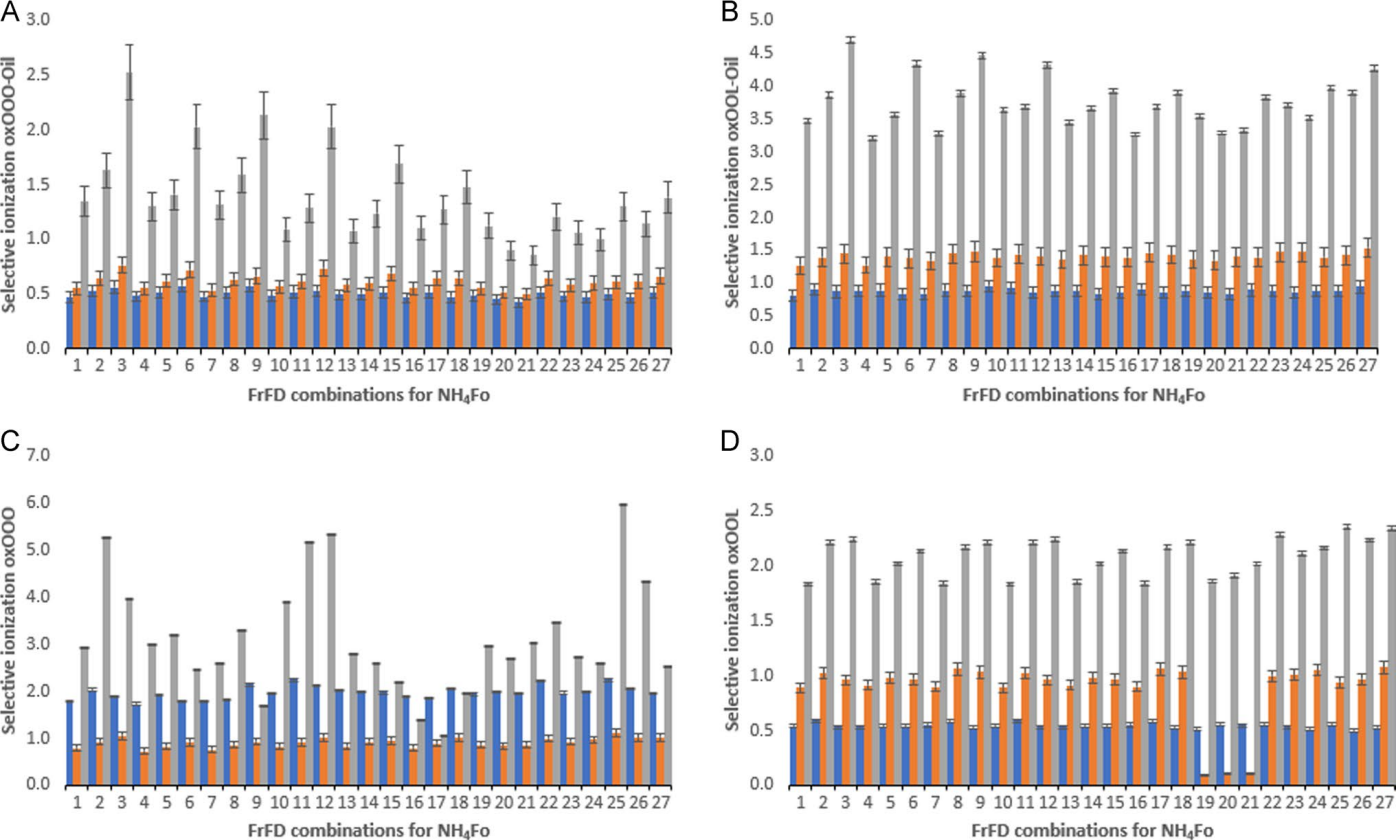
Selective ionization of oxidized versus non-oxidized TAG when using ammonium formate (NH_4_Fo) for two specific TAGs in standards and oxidized rapeseed oil: **A** OOO in rapeseed oil, **B** OOL in rapeseed oil standard, **C** OOO as standard compound, **D** OOL as standard compound. The *y*-axis depicts the selective ionization of specific TAG. The *x*-axis depicts the 27 combinations (#1–27) of the fractional factorial design (FrFD) for NH_4_Fo (see details in Supplemental Table S1). For each type of TAG, three types of oxTAGs were monitored: 1ox (blue), 2ox (orange), and 3ox (grey). Data shown are the averages measurements ± SD

Ammonium formate is widely used as a solvent additive for the ionization of lipids in both LC–MS and direct inlet MS [13, 14]. In our experiments, its use as an additive resulted in a high abundance of ammoniated adducts, albeit that also some protonated and sodiated ions were still seen. The selectivity values obtained for OOO and OOL (calculated based on ammoniated adducts), either measured as pure standards or from oxidized rapeseed oil, are presented (Fig. 3). Twenty-seven different condition sets were evaluated for eight different (oxidized) species, OOO, and OOL and their corresponding 1ox, 2ox, and 3ox TAGs. These experiments were done in oxidized oil (Fig. 3A and B) as well as in pure standards (Fig. 3C and D) to detect possible ionization suppression effects in real samples. Again, next to relative sensitivities, the values also reflect errors caused by, e.g. specific fragments of oxidized species having the same mass as that selected for the non-oxidized species. If, for example, a single oxygen oxidized species would lose its oxygen upon ionization, this species would be registered as a non-oxidized compound.

For the oxidized OOO and OOL standards, the overall highest selectivity was obtained at condition set 25 (0.2 mM NH_4_Fo, 350 °C, 2000 V capillary voltage, and 1000 V nozzle voltage) as shown in Fig. 3C and D, although the ionization parameters had a greater influence on the selectivity for the oxidized OOO standard compared to the OOL one. On the other hand, for the oxidized OOO and OOL in the rapeseed oil samples, the highest selectivity was obtained with condition set 3 (0.05 mM NH_4_Fo, 150 °C, 5000 V capillary voltage, and 1500 V nozzle voltage). When looking to the influence of ion source parameters, it is clear that the selectivity values followed more or less the same trend, with a more pronounced effect for the 3ox TAG. The ionization parameters had a relatively limited influence for these ammoniated adducts. For several combinations of analytes and settings, selective ionization factors below 1 were observed, indicating that ionization of non-oxidized TAGs was favored over that for oxidized TAG, which is the opposite of what we intended to achieve. This is for example the case for the 1ox-OOL standard or the 1ox OOO and 2ox OOO in the oxidized rapeseed oil sample. On the other hand, for 2ox-OOL in oil, the selectivity factor is around 1.4, showing that here ionization of 2ox-OOL is slightly favored. Anyhow, it is clear from the data that the selectivity values obtained for this additive are rather limited, with a maximum value only around 6. If we assume mass selectivity to be infinite, i.e., no oxidized species or fragments with the same mass as the non-oxidized compound and vice versa, this indicates that the 3ox-OOO ionizes 6 times better than the non-oxidized OOO. It is also important to note that the selectivity values measured using pure standards differ from those obtained from oils. As an example, for the 1ox-OOO standard, most selected ionization settings favored the oxidized species, whereas in oils the ionization of the non-oxidized OOO was favored. Similarly, the selectivity for all oxidized OOO species was lower in a real oil sample compared to a pure standard, whereas it was higher for the oxidized OOL species. These differences in response between oil and in the standards could either result from matrix effects, i.e., competition for ionization, but could also result from the assumption made in the calculations. Our semi-quantitative calculations were based on total hydroperoxides, total aldehydes, and total epoxides, leading to the assumption that all TAG-fatty acid chains oxidized the same way, which is not the case in reality. Oxidation rates for OOL will be higher than for OOO. To summarize the findings, in terms of sensitivity ammonium formate performed rather well with a good sensitivity for both oxidized and non-oxidized species, but in terms of selectivity, its performance was disappointing.

#### Sodium-containing additives

**Fig. 4:**
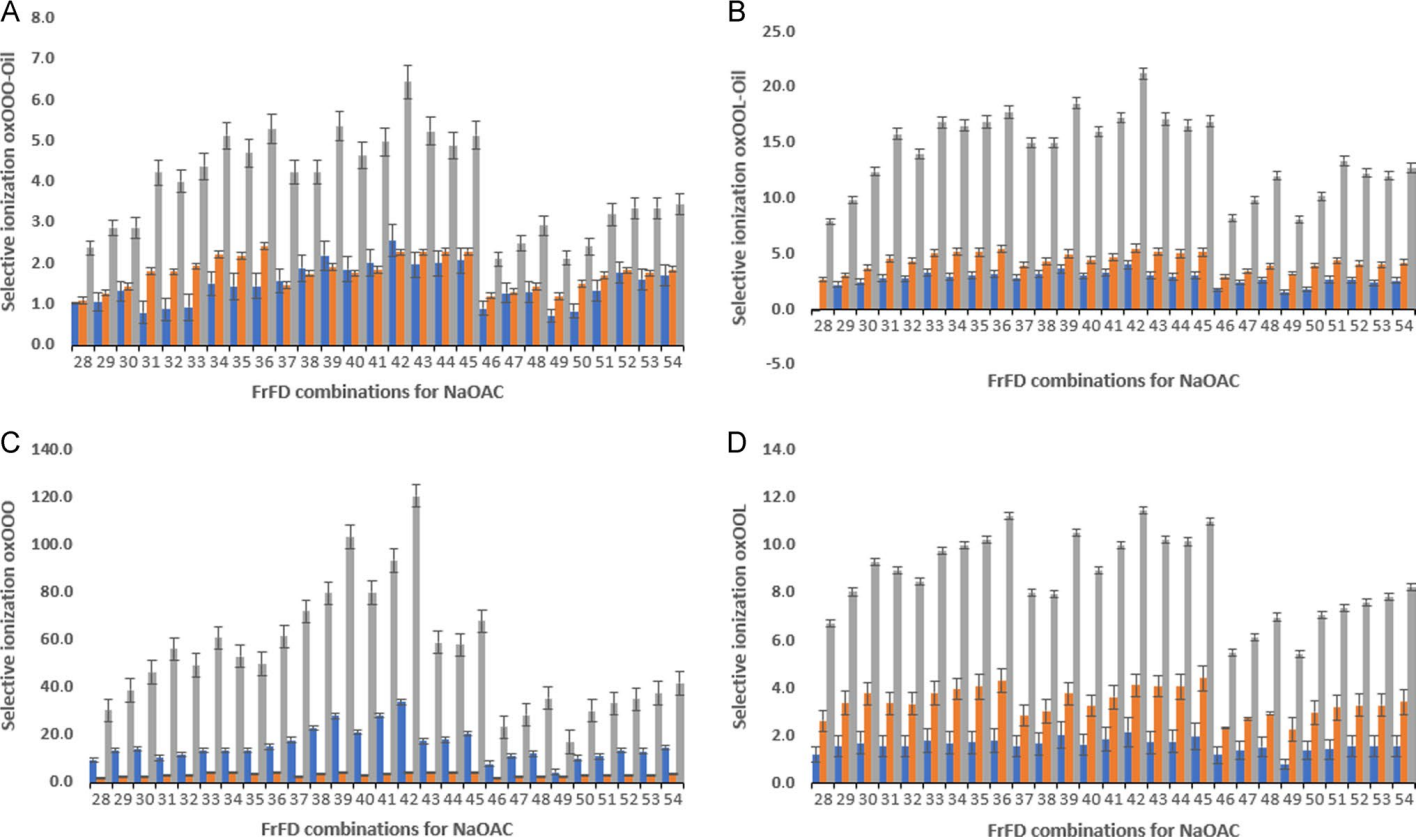
Selective ionization of oxidized versus non-oxidized TAG when using sodium acetate (NaOAc) for two specific TAGs in standards and oxidized rapeseed oil: **A** OOO in rapeseed oil, **B** OOL in rapeseed oil, **C** OOO as standard compound, **D** OOL as standard compound. The *y*-axis depicts the selective ionization of specific TAG. The *x*-axis depicts the 27 combinations (#28–54) of the fractional factorial design (FrFD) for NaOAc (see details in Supplemental Table S1). For each type of TAG, three types of oxTAGs were monitored: 1ox (blue), 2ox (orange), and 3ox (grey). Data shown are the averaged measurements ± SD

The selectivity of ionization when using sodium-containing additives was evaluated in a similar manner as for the ammonium formate additive, now monitoring sodiated adduct ions instead of the ammoniated species. As observed in Figs. 3 and 4, sodium-containing additives (NaOAc and NaI, respectively) gave much higher overall ionization selectivities as compared to ammonium containing additives (Fig. 2). This was in line with the results obtained at the first stage of investigation where mass windows instead of specific masses were monitored.

When using NaOAc as the additive, conditions set # 42 (0.1 mM NaOAc; 250 °C sheath gas temperature; 5000 V capillary voltage, and 1500 V nozzle voltage) provided the highest selectivity both for OOO and OOL, in both standards and real oil (Fig. 3). With this additive, the ionization selectivity for OOO from the standard solution was as high as 120 for 3ox-OOO, 34 for 1ox-OOO, and 4.5 for 2ox-OOO (Fig. 3C). These values are considerable improvements relative to those obtained using ammonium formate as the mobile phase additive where the maximum found for 3ox OOO was only around 6. Whereas one would expect 2ox-OOO to take an intermediate position between 1ox- and 3ox-OOO in terms of ionization selectivity, this is not what was found in the experiments. It could be that 2ox-OOO oxidation products, most likely hydroperoxides, are less stable, especially under harsher ionization conditions. The corresponding values for the OOL standard were 11.5, 4.4, and 2.1 for 1ox-OOL, 2ox-OOL, and 3ox-OOL, respectively (Fig. 3D). The lower values obtained for this analyte are most likely not a result of the poorer ionization of the oxidized species, but rather of the easier ionization of the non-oxidized OOL. Due to the higher number of double bonds present in the non-oxidized OOL versus the non-oxidized OOO, the first species is easier ionized.

Similar to the situation with NH_4_Fo addition, the ionization selectivities for OOO and OOL standards were significantly different when compared to the values found for the oil samples. On the other hand, the optimum settings did not differ. Again, the selectivity for OOO in a real oil sample was found to be lower as compared to that for the pure compounds, whereas for the oxidized OOL again higher values were found in the real oil. This probably reflects the above average oxidation and ionization rates of OOL in a real oil sample vs. the lower-than-average corresponding rates for OOO in the real oil.

**Fig. 5:**
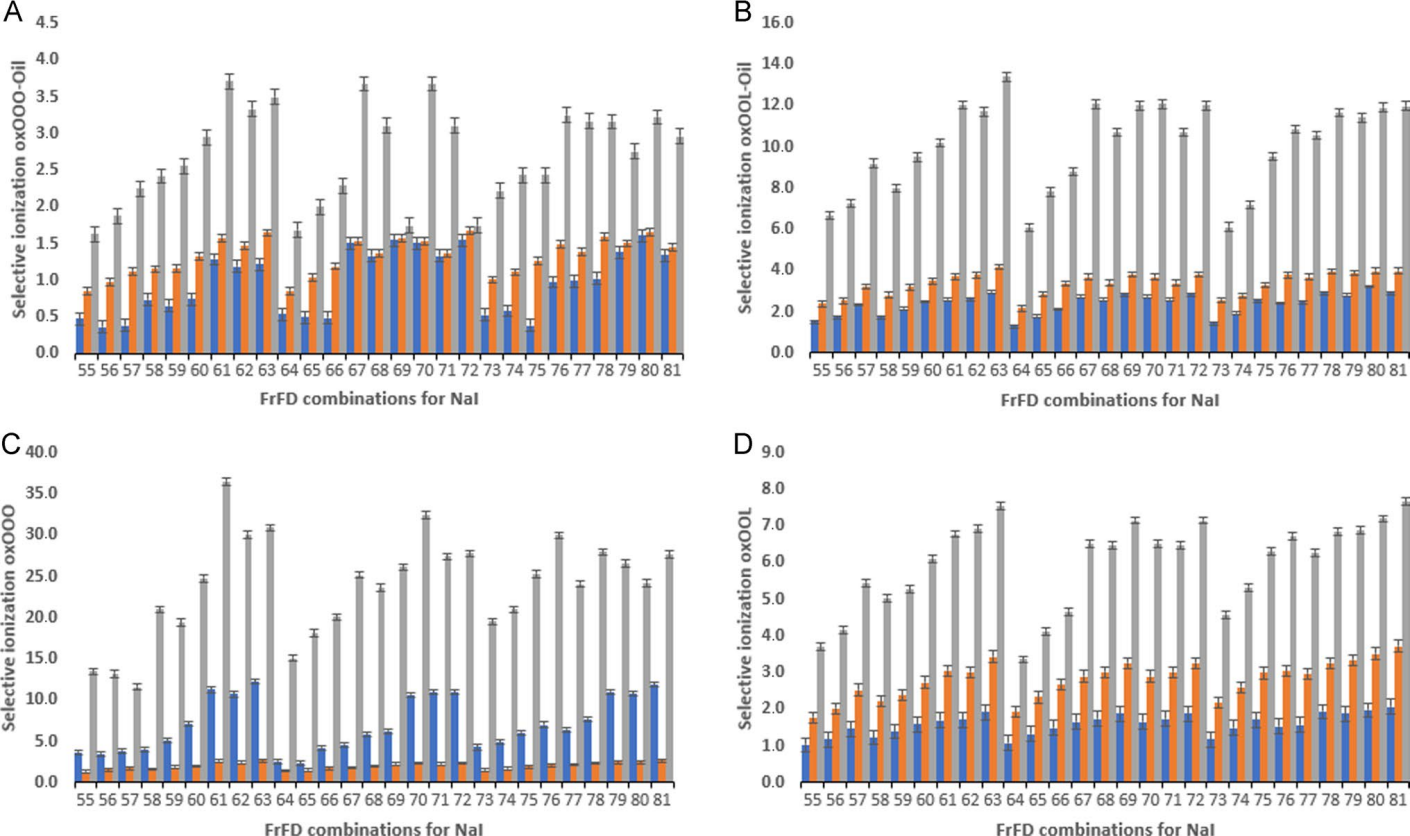
Selective ionization of oxidized versus non-oxidized TAG when using sodium iodide (NaI) for two specific triacylglycerols (TAGs) in standards and oxidized rapeseed oil: **A** OOO in rapeseed oil, **B** OOL in rapeseed oil, **C** OOO standard, **D** OOL standard. The *y*-axis depicts the selective ionization the specific TAG. The *x*-axis depicts the 27 combinations (#55–81) of the fractional factorial design (FrFD) for NaI (see details in Supplemental Table S1). For each type of TAG, three types of oxTAGs were monitored: 1ox (blue), 2ox (orange), and 3ox (grey). Data shown are the averaged measurements ± SD

The sodiated ion results discussed above were obtained using NaOAc as the additive. From a comparison of Figs. 4 and 5, it is clear that the selectivity pattern (i.e., influence of ionization settings) is largely similar for all oxidized species studied, irrespective of whether NaOAc or NaI is used, albeit that the absolute selectivity values are somewhat higher for NaOAc. Moreover, when using NaI as solvent additive, no clear optimum condition set exists. These optimum conditions could lay somewhere within the range of parameters tested in the FrFD*.* Here the types of lipids considered as well as the type of oxidized products monitored do affect the optimum ionization selectivity, meaning that in this case there is no single set of conditions that provides the highest selectivities for all TAG simultaneously. Overall, the most promising results when using NaI were achieved when applying conditions # 63 (0.05 mM NaI; 350 °C sheath gas temperature; 5000 V capillary voltage; and 500 V nozzle voltage). Why optimum settings for the two sodium salts are different is unclear. When looking at the bars corresponding to 1, 2, and 3ox TAGs in oil for both sodium-containing additives (NaOAc and NaI), it is clear that they follow a similar trend as encountered when using NH_4_Fo, with the influence of ion source settings on selectivity being more pronounced for 3ox TAG compared to 1ox and 2ox species. For the 1ox OOO and 2ox OOO in the oxidized rapeseed oil sample, the ionization selectivity factors found ranged from 0.5 and 2.5 at all conditions tested for NaOAc, and from 0.4 to 1.5 for NaI, all rather disappointing values. For 1ox and 2ox-OOL in oil, slightly higher selectivity factors were found reaching values up to 5.5 and 4.2, when using NaOAc and NaI, respectively. Similar to the findings with NH_4_Fo, the ionization of 1 and 2ox-OOL was favored relative to OOO. Addition of NaOAc as a solvent additive increased the ionization selectivity to 6.4 for 3ox-OOO or 21.2 for 3ox-OOL (Fig. 3A and B), whereas addition of NaI only results in selectivity values up to 3.7 and 13.4 (Fig. 4A and B). Possibly the acetate or iodide counter ions influence the solvent pH which in turn will affect the stability of the adduct complex and hence the ionization efficiency [25]. Again, the exact reason is unclear, yet it is evident that careful selection of the ionization conditions is important for maximum selectivity and ruggedness of the method.

As already indicated above, for NaOAc, one set of conditions showed the highest ionization efficiency for all oxidized lipid species as standards or from a real oil (i.e., 0.1 mM NaOAc; 250 °C sheath gas temperature; 5000 V capillary voltage; and 1500 V nozzle voltage). The fact that one set of conditions results in the best performance for all target species of course is highly attractive from a practical perspective and should be considered when selecting the ionization conditions.

Overall, the second stage of data investigation that focusses on specific masses shows an improved performance regarding the ability to selectively detect oxidized lipid species vs. their non-oxidized counterparts. For real oil samples, selectivity factors as high as 25 can be obtained under optimum conditions as compared to factors below 0.5 for poorly chosen settings. For pure standards, selectivity values as high as 120 were obtained. Gaining more insights into the contribution of individual ionization factors and their interaction is therefore essential and will be discussed in the next section.

### Selection of optimal factor combinations and validation

Although visual inspection of the graphs discussed above does provide an indication on the combination of factors providing the highest selectivity, it does not immediately provide clear insights regarding which individual factors are the most relevant for providing a high selectivity. One point to emphasize is that statistical significance was so far not considered and other condition sets (i.e., not experimentally tested yet in the FrFD) might also provide a performance that is statistically significantly better or worse. The fractional factorial design (FrFD) can contribute more information regarding not only condition sets that provide the highest selectivity values, but also identify two-way significant interactions between factors that could affect the selective ionization of oxidized versus non-oxidized lipid species. Therefore, the results of the second stage investigation for all 81 combinations were introduced in the FrFD model. Unfortunately, the fractional factorial design did not have enough degrees of freedom to provide truly quantitative results, but it allows to derive qualitative recommendations.

To gain more insight regarding the influence of the 5 factors’ main effects and 10 two-way interactions on selective ionization, analysis of variance (ANOVA) was performed (SI-Table 3) followed by Tukey’s comparisons. The ANOVA results showed a significant main effect for all compounds of interest for “type of solvent additive” (A) and “concentration of solvent additive” (B). Furthermore, except for 3ox OOO from the OOO standard and 1ox and 3ox OOO in oxidized oil, “sheath gas temperature” (C) and “capillary voltage” (D), also significantly affected most compounds of interest, whereas “nozzle voltage” (E) had no significant effect. Several two-way interactions significantly contributed to changes in selective ionization (*p* < 0.05; SI-Table 4). More specifically, the most significant two-way interactions were observed for AB, AC, AD, BC, and CD. Interaction plots for these significant two-way interactions are shown in Supplementary Fig. 2 (Supplemental Material-Fig. 2). Depending on the compound of interest (either regarding type of TAG or degree of oxidation), the significance level changed, as did the variance explained from the two-way interactions. All in all, the ANOVA results highlighted that the best conditions to achieve a high selectivity were the use of 0.1 mM NaOAc with intermediate to high sheath gas temperature (250–350 °C) and high capillary voltage (5000 V), while the nozzle voltage did not have an influence.

The optimal conditions suggested by the Tukey´s comparisons corresponded to conditions set #42 from the FrFD (Supplementary material Table S1), which indeed provided the highest selectivities. To confirm the reproducibility of these results, the combination of optimum settings (NaOAc; 0.1 mM NaOAc; 250 °C sheath gas temperature; 5000 V capillary voltage; and 1000 or 1500 V nozzle voltage) were tested in triplicate. The validation results can be found in the Supplementary Material Table S5. By carefully optimizing the ionization conditions, our research showed that it is possible to enhance the selectivity of oxidized lipid species in MS analysis and make their quantification in food samples more reliable. Finally, it is important to emphasize that the findings of the current study can be applied both in high-throughput direct inlet or flow infusion MS food safety screening and in detailed LC–MS investigations of oxidized species in food samples.

## Conclusion

In this work, a novel direct infusion MS approach was developed for the selective ionization and detection of oxidized lipid species while suppressing the signals of their non-oxidized precursors. The combination of medium concentration NaOAc as solvent additive, with medium sheath gas temperature and high capillary voltage, successfully achieved an increased selective ionization for all investigated species. Moreover, the ANOVA results confirmed that levels of main factors influencing selectivity of oxidized versus non-oxidized compounds largely depend on the specific compounds of interest, but that a universal set of optimum conditions can be found. Clearly, the optimized parameters selected in this study provided a significant enhancement in selectivity of oxidized vs. non-oxidized TAGs. In itself this selectivity might not be sufficient for direct analysis of oxidized species, but application of the selective MS settings identified here will alleviate the requirements to be imposed on the sample preparation and separation stages of protocols for the analysis of oxidized lipids.

### Supplementary information

Below is the link to the electronic supplementary material.

Supplementary file1 (DOCX 1579 KB)
