# Supplementary material for: Selective ionization of oxidized lipid species using different solvent additives in flow injection mass spectrometry
Source: Anal Bioanal Chem. 2023 Oct 17;416(3):787–99. doi: 10.1007/s00216-023-04988-x (PMC10766781; doi:10.1007/s00216-023-04988-x)
Supplement: Supplementary file 1 — Supplementary file1 (DOCX 1579 KB) [file 216_2023_4988_MOESM1_ESM.docx]

**Supplementary material**

R script for the Fractional Factorial Design Experimental Design

#require(devtools)

#install_version("planor", version = "1.5.3")

library(planor)

library(car)

library(agricolae)

set.seed(123)

#define experimental factors and their levels

#A

SolventAdditive <- as.factor(c("Ammonium Formate", "Sodium Acetate", "Sodium Iodide"))

#B

ConcentrationSolventAdditive <- as.factor(c("Low", "Medium", "High"))

#C

SheathGasTemperature <- as.factor(c(150, 250, 350))

#D

CapillaryVoltage <- as.factor(c(2000, 3500, 5000))

#E

NozzleVoltage <- as.factor(c(500, 1000, 1500))

#create design

myData <- as.data.frame(regular.design(factors=LETTERS[1:5], nlevels=rep(3,5),

resolution=5, model=~(A+B+C+D+E)^2, nunits=3^4))

#substitute factor name and levels

colnames(myData)[1] <- 'SolventAdditive'

myData[,'SolventAdditive'] <- SolventAdditive[myData[,'SolventAdditive']]

colnames(myData)[2] <- 'ConcentrationSolventAdditive'

myData[,'ConcentrationSolventAdditive'] ConcentrationSolventAdditive[myData[,'ConcentrationSolventAdditive']]

colnames(myData)[3] <- 'SheathGasTemperature'

myData[,'SheathGasTemperature'] <- SheathGasTemperature[myData[,'SheathGasTemperature']]

colnames(myData)[4] <- 'CapillaryVoltage'

myData[,'CapillaryVoltage'] <- CapillaryVoltage[myData[,'CapillaryVoltage']]

colnames(myData)[5] <- 'NozzleVoltage'

myData[,'NozzleVoltage'] <- NozzleVoltage[myData[,'NozzleVoltage']]

head(myData)

R script for the statistical analysis

library(readxl)

library(car, quietly = T)

library(emmeans)

library(stringr)

library(ggplot2)

fprintf <- function(...) cat(sprintf(...))

output_to_pdf <- TRUE

output_to_text_file <- TRUE

dataFilename <- "SELECTIVE IONIZATION_JOS.xlsx"

sheets <- excel_sheets(dataFilename)

whichOx <- c("ox1", "ox2", "ox3")

#output all text from script to diary file

if (output_to_text_file) sink("Text Output for all analysis.txt")

#repeat all models etc for peak area and peak height

for (whichsheet in sheets) {

fprintf("Reading sheet: %s\n", whichsheet)

#read in the data

mydata <- suppressMessages(

as.data.frame(read_excel(dataFilename, sheet = whichsheet, col_names = TRUE, skip = 1)))

#adjust rownames

rownames(mydata) <- mydata[,1]

mydata <- mydata[,-1]

mydata <- mydata[,-1]

#adjust columnnames for easy modelling

colnames(mydata) <- c("Type", "Concentration", "Temperature", "Capillary", "Nozzle" ,"ox1", "ox2", "ox3")

#adjust concentration into low, medium, high

mydata$Concentration[mydata$Concentration==5 | mydata$Concentration==0.05] = "low"

mydata$Concentration[mydata$Concentration==10 | mydata$Concentration==0.1] = "medium"

mydata$Concentration[mydata$Concentration==20 | mydata$Concentration==0.2] = "high"

#turn all into factors

mydata$Type <- as.factor(mydata$Type)

mydata$Concentration <- factor(mydata$Concentration, levels=c("low","medium","high"))

mydata$Temperature <- as.factor(mydata$Temperature)

mydata$Capillary <- as.factor(mydata$Capillary)

mydata$Nozzle <- as.factor(mydata$Nozzle)

#print(head(mydata))

#end of reading in data

#repeat modelling and subsequent analysis for ox1, ox2 and ox3

for (s in whichOx) {

#save plots to pdf instead of printing to screen

if (output_to_pdf) {

pdfOutfname2 <- paste(whichsheet, s, "diagnostic and profile plots.pdf")

pdf(file=pdfOutfname2, paper='a4')

}

#correlations between ox1, ox2 and ox3 are all high

cor(mydata[,whichOx])

pairs(mydata[,whichOx])

title(whichsheet, line = -1, outer = TRUE)

#create model for ox?

MyModelFormula <- as.formula(paste(s ," ~ (Type + Concentration + Temperature + Capillary + Nozzle)^2"))

fprintf("Model: %s\n", format(MyModelFormula))

myModel <- lm(MyModelFormula, data=mydata)

MyAnova <- Anova(myModel, type=3)

print(MyAnova)

print(summary(myModel))

#check residuals

par(mfrow=c(1,3))

plot(myModel, c(1,2,5), ask=F)

title(format(MyModelFormula), line = -1, outer = TRUE)

par(mfrow=c(1,1))

#extract emmeans profile plots in an automated way

idx <- rownames(MyAnova)[MyAnova$`Pr(>F)` < 0.05][-1]

idx <- idx[!is.na(idx)]

#find sign 2way interactions

myInteractions <- grep(":", idx)

#get sign main effects

myMains <- setdiff(1:length(idx), myInteractions)

#get main effects not in interaction

tmp <- sapply(idx[myMains], function(i) {

sum(str_detect(idx, i))

})

if(sum(tmp)==0) print("Significant main effect without sign interaction found.") #this does not happen, so ignore

#do EMMEANS and profile plots for all significant interaction effects

for (i in 1:length(myInteractions)) {

myEmmeansFormula <- as.formula(paste("pairwise ~ ", gsub(":", "|", idx[myInteractions][i])))

myEmmipFormula <- as.formula(gsub(":", "~", idx[myInteractions][i]))

#give the numbers

p1 <- emmeans(myModel, myEmmeansFormula)

print(p1)

#make profile plot

p2 <- emmip(myModel, myEmmipFormula, CIs = TRUE) +

ggtitle(format(MyModelFormula))

print(p2)

}

#if output to file, close the file

if (output_to_pdf) dev.off()

#make plot of all treatments and CIs

tmp <- emmeans(myModel, ~ (Type + Concentration + Temperature + Capillary + Nozzle)^2)

#open new file is desired, this pdf is very large to hold big figure

if (output_to_pdf) {

pdfOutfname <- paste(whichsheet, s, "Overview of treatments.pdf")

pdf(file=pdfOutfname, width = 20, height = 75)

}

#do pairwise on all treatments

#res <- pairs(tmp) #because of huge number of treatments this is not feasible

#instead do overview plot with CIs

#make overview with CI for all treatments

p3 <- plot(tmp, plotit=TRUE) + ggtitle(format(MyModelFormula))

print(p3)

#close the file if needed

if (output_to_pdf) dev.off()

}

}

#close text output file if needed

if (output_to_text_file) sink()

Table S1 Fractional factorial design (FrFD) combinations of factors and their levels. Factor A represents the solvent additives with 1 corresponding to ammonium formate, 2 to sodium acetate, and 3 to sodium iodide. Factor B represents the solvent additive concentration. For ammonium formate, levels 1,2 and 3 correspond to 5, 10 and 20 mM, respectively. For sodium acetate and sodium iodide, levels 1,2 and 3 correspond to 0.05, 0.1 and 0.2 mM respectively. Factor C represents sheath gas temperature with levels 1,2 and 3 corresponding to 150, 250 and 350 ^o^C. Factor D represents capillary voltage with levels 1,2 and 3 corresponding to 2000, 3500 and 5000 V. Factor E represents nozzle voltage with levels 1,2 and 3 corresponding to 500, 1000 and 1500 V.

| **FrFD conditions** | **Factor levels** | | | | | **FrFD conditions** | **Factor levels** | | | | | **FrFD conditions** | **Factor levels** | | | | |
| --- | --- | --- | --- | --- | --- | --- | --- | --- | --- | --- | --- | --- | --- | --- | --- | --- | --- |
|  | **A** | **B** | **C** | **D** | **E** |  | **A** | **B** | **C** | **D** | **E** |  | **A** | **B** | **C** | **D** | **E** |
| **1** | **1** | **1** | **1** | **1** | **1** | **28** | **2** | **1** | **1** | **1** | **2** | **55** | **3** | **1** | **1** | **1** | **3** |
| **2** | **1** | **1** | **1** | **2** | **2** | **29** | **2** | **1** | **1** | **2** | **3** | **56** | **3** | **1** | **1** | **2** | **1** |
| **3** | **1** | **1** | **1** | **3** | **3** | **30** | **2** | **1** | **1** | **3** | **1** | **57** | **3** | **1** | **1** | **3** | **2** |
| **4** | **1** | **1** | **2** | **1** | **2** | **31** | **2** | **1** | **2** | **1** | **3** | **58** | **3** | **1** | **2** | **1** | **1** |
| **5** | **1** | **1** | **2** | **2** | **3** | **32** | **2** | **1** | **2** | **2** | **1** | **59** | **3** | **1** | **2** | **2** | **2** |
| **6** | **1** | **1** | **2** | **3** | **1** | **33** | **2** | **1** | **2** | **3** | **2** | **60** | **3** | **1** | **2** | **3** | **3** |
| **7** | **1** | **1** | **3** | **1** | **3** | **34** | **2** | **1** | **3** | **1** | **1** | **61** | **3** | **1** | **3** | **1** | **2** |
| **8** | **1** | **1** | **3** | **2** | **1** | **35** | **2** | **1** | **3** | **2** | **2** | **62** | **3** | **1** | **3** | **2** | **3** |
| **9** | **1** | **2** | **3** | **3** | **2** | **36** | **2** | **2** | **3** | **3** | **3** | **63** | **3** | **2** | **3** | **3** | **1** |
| **10** | **1** | **2** | **1** | **1** | **2** | **37** | **2** | **2** | **1** | **1** | **3** | **64** | **3** | **2** | **1** | **1** | **1** |
| **11** | **1** | **2** | **1** | **2** | **3** | **38** | **2** | **2** | **1** | **2** | **1** | **65** | **3** | **2** | **1** | **2** | **2** |
| **12** | **1** | **2** | **1** | **3** | **1** | **39** | **2** | **2** | **1** | **3** | **2** | **66** | **3** | **2** | **1** | **3** | **3** |
| **13** | **1** | **2** | **2** | **1** | **3** | **40** | **2** | **2** | **2** | **1** | **1** | **67** | **3** | **2** | **2** | **1** | **2** |
| **14** | **1** | **2** | **2** | **2** | **1** | **41** | **2** | **2** | **2** | **2** | **2** | **68** | **3** | **2** | **2** | **2** | **3** |
| **15** | **1** | **2** | **2** | **3** | **2** | **42** | **2** | **2** | **2** | **3** | **3** | **69** | **3** | **2** | **2** | **3** | **1** |
| **16** | **1** | **2** | **3** | **1** | **1** | **43** | **2** | **2** | **3** | **1** | **2** | **70** | **3** | **2** | **3** | **1** | **3** |
| **17** | **1** | **2** | **3** | **2** | **2** | **44** | **2** | **2** | **3** | **2** | **3** | **71** | **3** | **2** | **3** | **2** | **1** |
| **18** | **1** | **3** | **3** | **3** | **3** | **45** | **2** | **3** | **3** | **3** | **1** | **72** | **3** | **3** | **3** | **3** | **2** |
| **19** | **1** | **3** | **1** | **1** | **3** | **46** | **2** | **3** | **1** | **1** | **1** | **73** | **3** | **3** | **1** | **1** | **2** |
| **20** | **1** | **3** | **1** | **2** | **1** | **47** | **2** | **3** | **1** | **2** | **2** | **74** | **3** | **3** | **1** | **2** | **3** |
| **21** | **1** | **3** | **1** | **3** | **2** | **48** | **2** | **3** | **1** | **3** | **3** | **75** | **3** | **3** | **1** | **3** | **1** |
| **22** | **1** | **3** | **2** | **1** | **1** | **49** | **2** | **3** | **2** | **1** | **2** | **76** | **3** | **3** | **2** | **1** | **3** |
| **23** | **1** | **3** | **2** | **2** | **2** | **50** | **2** | **3** | **2** | **2** | **3** | **77** | **3** | **3** | **2** | **2** | **1** |
| **24** | **1** | **3** | **2** | **3** | **3** | **51** | **2** | **3** | **2** | **3** | **1** | **78** | **3** | **3** | **2** | **3** | **2** |
| **25** | **1** | **3** | **3** | **1** | **2** | **52** | **2** | **3** | **3** | **1** | **3** | **79** | **3** | **3** | **3** | **1** | **1** |
| **26** | **1** | **3** | **3** | **2** | **3** | **53** | **2** | **3** | **3** | **2** | **1** | **80** | **3** | **3** | **3** | **2** | **2** |
| **27** | **1** | **3** | **3** | **3** | **1** | **54** | **2** | **3** | **3** | **3** | **2** | **81** | **3** | **3** | **3** | **3** | **3** |

Table S2 Probability calculations based on NMR quantification.

|  |  |  | **OOO** | | **OOL** | | **Oxidized rapeseed oil** | |
| --- | --- | --- | --- | --- | --- | --- | --- | --- |
|  |  | **Non-Ox** | 0,971 | | 0,799 | | 0,874 | |
|  |  | **1ox** | 0,003 | | 0,074 | | 0,056 | |
|  |  | **2ox** | 0,026 | | 0,127 | | 0,07 | |
| **Combinations** | **FA positions** | **# Of Ox** |  | **CHANCE** |  | **CHANCE** |  | **CHANCE** |
| **1** | 0-0-0 | 0 | 0,915499 | 91,550 | 0,510 | 51,008 | 0,668 | 66,763 |
| **2** | 0-0-1 | 1 | 0,002829 |  | 0,047 |  | 0,043 |  |
| **3** | 0-1-0 | 1 | 0,002829 |  | 0,047 |  | 0,043 |  |
| **4** | 1-0-0 | 1 | 0,002829 | 0,849 | 0,047 | 14,173 | 0,043 | 12,833 |
| **5** | 1-1-0 | 2 | 0,000009 |  | 0,004 |  | 0,003 |  |
| **6** | 1-0-1 | 2 | 0,000009 |  | 0,004 |  | 0,003 |  |
| **7** | 0-1-1 | 2 | 0,000009 |  | 0,004 |  | 0,003 |  |
| **8** | 0-0-2 | 2 | 0,024514 |  | 0,081 |  | 0,053 |  |
| **9** | 0-2-0 | 2 | 0,024514 |  | 0,081 |  | 0,053 |  |
| **10** | 0-0-2 | 2 | 0,024514 | 7,357 | 0,081 | 25,636 | 0,053 | 16,864 |
| **11** | 1-1-1 | 3 | 0,000000 |  | 0,000 |  | 0,000 |  |
| **12** | 0-1-2 | 3 | 0,000076 |  | 0,008 |  | 0,003 |  |
| **13** | 0-2-1 | 3 | 0,000076 |  | 0,008 |  | 0,003 |  |
| **14** | 1-0-2 | 3 | 0,000076 |  | 0,008 |  | 0,003 |  |
| **15** | 1-2-0 | 3 | 0,000076 |  | 0,008 |  | 0,003 |  |
| **16** | 2-0-1 | 3 | 0,000076 |  | 0,008 |  | 0,003 |  |
| **17** | 2-1-0 | 3 | 0,000076 | 0,045 | 0,008 | 4,546 | 0,003 | 2,073 |
| **18** | 0-2-2 | 4 | 0,000656 |  | 0,013 |  | 0,004 |  |
| **19** | 2-0-2 | 4 | 0,000656 |  | 0,013 |  | 0,004 |  |
| **20** | 2-2-0 | 4 | 0,000656 |  | 0,013 |  | 0,004 |  |
| **21** | 1-1-2 | 4 | 0,000000 |  | 0,001 |  | 0,0002 |  |
| **22** | 1-2-1 | 4 | 0,000000 |  | 0,001 |  | 0,0002 |  |
| **23** | 2-1-1 | 4 | 0,000000 | 0,197 | 0,001 | 4,075 | 0,0002 | 1,351 |
| **24** | 2-2-1 | 5 | 0,000002 |  | 0,001 |  | 0,0003 |  |
| **25** | 2-1-2 | 5 | 0,000002 |  | 0,001 |  | 0,0003 |  |
| **26** | 1-2-2 | 5 | 0,000002 | 0,001 | 0,001 | 0,358 | 0,0003 | 0,082 |
| **27** | 2-2-2 | 6 | 0,000018 | 0,002 | 0,002 | 0,205 | 0,0003 | 0,034 |
| **Sum** |  |  | 1 | 100 | 1 | 100 | 1 | 100 |

Table S3. Selective ionization for each compound of interest tested for all fractional factorial design conditions at the first and second stage of investigation. A. OOO B. OOL C. Rapeseed Oil D. 1oxOOO E. 2oxOOO F. 3oxOOO G. 1oxOOL H. 2oxOOL I. 3oxOOL J. 1oxOOO in rapeseed oil K. 2oxOOO in rapeseed oil L. 3oxOOO in rapeseed oil M. 1oxOOL in rapeseed oil N. 2oxOOL in rapeseed oil O 3oxOOL in rapeseed oil.

|  | **First Stage Investigation** | | | **Second Stage**  **Investigation** | | | | | | | | | | | |
| --- | --- | --- | --- | --- | --- | --- | --- | --- | --- | --- | --- | --- | --- | --- | --- |
| **#** | **A** | **B** | **C** | **D** | **E** | **F** | **G** | **H** | **I** | **J** | **K** | **L** | **M** | **N** | **O** |
| **1** | 4,3 | 1,7 | 1,5 | 1,8 | 0,8 | 2,9 | 0,5 | 0,9 | 1,8 | 0,5 | 0,6 | 1,3 | 0,8 | 1,3 | 3,5 |
| **2** | 3,9 | 1,7 | 1,6 | 2,0 | 0,9 | 5,3 | 0,6 | 1,0 | 2,2 | 0,5 | 0,6 | 1,6 | 0,9 | 1,4 | 3,9 |
| **3** | 3,7 | 1,7 | 1,6 | 1,9 | 1,0 | 4,0 | 0,5 | 1,0 | 2,2 | 0,6 | 0,8 | 2,5 | 0,9 | 1,4 | 4,7 |
| **4** | 4,4 | 1,7 | 1,7 | 1,7 | 0,7 | 3,0 | 0,5 | 0,9 | 1,9 | 0,5 | 0,6 | 1,3 | 0,9 | 1,3 | 3,2 |
| **5** | 3,6 | 1,7 | 1,7 | 1,9 | 0,8 | 3,2 | 0,5 | 1,0 | 2,0 | 0,5 | 0,6 | 1,4 | 0,9 | 1,4 | 3,6 |
| **6** | 3,5 | 1,9 | 1,8 | 1,8 | 0,9 | 2,4 | 0,5 | 1,0 | 2,1 | 0,6 | 0,7 | 2,0 | 0,8 | 1,4 | 4,3 |
| **7** | 4,9 | 2,1 | 2,0 | 1,8 | 0,8 | 2,6 | 0,5 | 0,9 | 1,8 | 0,5 | 0,5 | 1,3 | 0,8 | 1,3 | 3,3 |
| **8** | 4,2 | 2,0 | 2,0 | 1,8 | 0,8 | 3,3 | 0,6 | 1,1 | 2,2 | 0,5 | 0,6 | 1,6 | 0,9 | 1,4 | 3,9 |
| **9** | 4,2 | 2,0 | 2,0 | 2,1 | 0,9 | 1,7 | 0,5 | 1,0 | 2,2 | 0,6 | 0,7 | 2,1 | 0,9 | 1,5 | 4,5 |
| **10** | 3,3 | 1,5 | 1,5 | 1,9 | 0,8 | 3,9 | 0,5 | 0,9 | 1,8 | 0,5 | 0,6 | 1,1 | 1,0 | 1,4 | 3,6 |
| **11** | 2,6 | 1,4 | 1,5 | 2,2 | 0,9 | 5,2 | 0,6 | 1,0 | 2,2 | 0,5 | 0,6 | 1,3 | 0,9 | 1,4 | 3,7 |
| **12** | 2,7 | 1,5 | 1,5 | 2,1 | 1,0 | 5,3 | 0,5 | 1,0 | 2,2 | 0,5 | 0,7 | 2,0 | 0,8 | 1,4 | 4,3 |
| **13** | 3,3 | 1,5 | 1,6 | 2,0 | 0,8 | 2,8 | 0,5 | 0,9 | 1,9 | 0,5 | 0,6 | 1,1 | 0,9 | 1,4 | 3,4 |
| **14** | 2,8 | 1,5 | 1,6 | 2,0 | 0,9 | 2,6 | 0,5 | 1,0 | 2,0 | 0,5 | 0,6 | 1,2 | 0,9 | 1,4 | 3,7 |
| **15** | 2,6 | 1,6 | 1,6 | 2,0 | 0,9 | 2,2 | 0,5 | 1,0 | 2,1 | 0,5 | 0,7 | 1,7 | 0,8 | 1,4 | 3,9 |
| **16** | 3,9 | 1,8 | 1,9 | 1,9 | 0,8 | 1,4 | 0,5 | 0,9 | 1,8 | 0,5 | 0,6 | 1,1 | 0,9 | 1,4 | 3,3 |
| **17** | 3,0 | 1,7 | 1,7 | 1,8 | 0,9 | 1,1 | 0,6 | 1,1 | 2,2 | 0,5 | 0,6 | 1,3 | 0,9 | 1,5 | 3,7 |
| **18** | 2,8 | 1,7 | 1,7 | 2,0 | 1,0 | 2,0 | 0,5 | 1,0 | 2,2 | 0,5 | 0,6 | 1,5 | 0,9 | 1,4 | 3,9 |
| **19** | 3,3 | 1,5 | 1,5 | 1,9 | 0,8 | 3,0 | 0,5 | 0,1 | 1,9 | 0,5 | 0,6 | 1,1 | 0,9 | 1,4 | 3,5 |
| **20** | 2,7 | 1,4 | 1,5 | 2,0 | 0,8 | 2,7 | 0,5 | 0,1 | 1,9 | 0,4 | 0,5 | 0,9 | 0,9 | 1,3 | 3,3 |
| **21** | 2,6 | 1,4 | 1,5 | 1,9 | 0,8 | 3,0 | 0,5 | 0,1 | 2,0 | 0,4 | 0,5 | 0,9 | 0,8 | 1,4 | 3,3 |
| **22** | 3,3 | 1,5 | 1,6 | 2,2 | 1,0 | 3,5 | 0,6 | 1,0 | 2,3 | 0,5 | 0,6 | 1,2 | 0,9 | 1,4 | 3,8 |
| **23** | 2,6 | 1,4 | 1,6 | 2,0 | 0,9 | 2,7 | 0,5 | 1,0 | 2,1 | 0,5 | 0,6 | 1,1 | 0,9 | 1,5 | 3,7 |
| **24** | 2,4 | 1,4 | 1,5 | 2,0 | 1,0 | 2,6 | 0,5 | 1,0 | 2,2 | 0,5 | 0,6 | 1,0 | 0,9 | 1,5 | 3,5 |
| **25** | 3,5 | 1,5 | 1,7 | 2,2 | 1,1 | 6,0 | 0,6 | 0,9 | 2,4 | 0,5 | 0,6 | 1,3 | 0,9 | 1,4 | 4,0 |
| **26** | 2,7 | 1,4 | 1,6 | 2,0 | 1,0 | 4,3 | 0,5 | 1,0 | 2,2 | 0,5 | 0,6 | 1,1 | 0,9 | 1,4 | 3,9 |
| **27** | 3,1 | 1,6 | 1,8 | 1,9 | 1,0 | 2,5 | 0,5 | 1,1 | 2,3 | 0,5 | 0,7 | 1,4 | 0,9 | 1,5 | 4,3 |
| **28** | 4,8 | 4,1 | 3,1 | 9,5 | 2,0 | 30,2 | 1,2 | 2,6 | 6,7 | 1,0 | 1,1 | 2,4 | 1,9 | 2,7 | 7,9 |
| **29** | 6,9 | 5,9 | 3,5 | 13,4 | 2,4 | 38,8 | 1,5 | 3,4 | 8,1 | 1,1 | 1,3 | 2,9 | 2,2 | 3,0 | 9,9 |
| **30** | 8,1 | 6,5 | 3,8 | 14,0 | 2,8 | 46,3 | 1,7 | 3,8 | 9,3 | 1,3 | 1,4 | 2,9 | 2,5 | 3,7 | 12,4 |
| **31** | 2,9 | 4,7 | 3,7 | 10,4 | 3,1 | 56,2 | 1,5 | 3,4 | 8,9 | 0,8 | 1,8 | 4,2 | 2,8 | 4,5 | 15,8 |
| **32** | 8,9 | 4,5 | 3,4 | 11,8 | 3,4 | 49,3 | 1,5 | 3,3 | 8,5 | 0,9 | 1,8 | 4,0 | 2,8 | 4,4 | 14,0 |
| **33** | 7,8 | 5,1 | 3,8 | 13,6 | 4,0 | 60,8 | 1,8 | 3,8 | 9,8 | 0,9 | 1,9 | 4,4 | 3,3 | 5,1 | 16,9 |
| **34** | 8,1 | 5,1 | 3,9 | 13,5 | 4,3 | 53,1 | 1,7 | 3,9 | 10,0 | 1,5 | 2,2 | 5,1 | 3,0 | 5,2 | 16,5 |
| **35** | 8,0 | 5,4 | 3,9 | 13,6 | 3,9 | 49,8 | 1,7 | 4,1 | 10,2 | 1,4 | 2,2 | 4,7 | 3,1 | 5,1 | 16,9 |
| **36** | 9,2 | 6,0 | 4,1 | 15,0 | 4,5 | 61,5 | 1,8 | 4,3 | 11,2 | 1,5 | 2,4 | 5,3 | 3,2 | 5,4 | 17,8 |
| **37** | 6,9 | 4,4 | 3,6 | 17,8 | 2,7 | 72,0 | 1,6 | 2,8 | 8,0 | 1,6 | 1,5 | 4,2 | 2,8 | 4,0 | 15,0 |
| **38** | 8,7 | 4,8 | 3,8 | 22,8 | 3,5 | 79,6 | 1,6 | 3,0 | 8,0 | 1,9 | 1,8 | 4,2 | 3,2 | 4,3 | 15,0 |
| **39** | 10,4 | 5,7 | 4,2 | 27,9 | 4,2 | 103,2 | 2,0 | 3,8 | 10,6 | 2,2 | 1,9 | 5,4 | 3,6 | 5,0 | 18,6 |
| **40** | 8,8 | 4,9 | 3,9 | 21,0 | 3,2 | 79,9 | 1,6 | 3,3 | 8,9 | 1,9 | 1,8 | 4,6 | 3,0 | 4,4 | 16,1 |
| **41** | 10,9 | 3,0 | 4,1 | 28,0 | 3,7 | 93,4 | 1,8 | 3,6 | 10,0 | 2,0 | 1,8 | 5,0 | 3,3 | 4,7 | 17,3 |
| **42** | 13,0 | 6,3 | 4,5 | 33,8 | 4,4 | 120,5 | 2,1 | 4,1 | 11,5 | 2,6 | 2,3 | 6,4 | 4,0 | 5,5 | 21,2 |
| **43** | 9,8 | 6,0 | 4,0 | 17,3 | 4,4 | 58,9 | 1,7 | 4,1 | 10,2 | 2,0 | 2,3 | 5,2 | 3,1 | 5,2 | 17,1 |
| **44** | 9,4 | 5,8 | 3,9 | 18,0 | 4,1 | 57,9 | 1,8 | 4,1 | 10,2 | 2,0 | 2,3 | 4,9 | 3,0 | 5,0 | 16,5 |
| **45** | 11,0 | 6,2 | 4,1 | 20,4 | 4,4 | 67,9 | 2,0 | 4,4 | 11,0 | 2,1 | 2,3 | 5,1 | 3,1 | 5,1 | 16,9 |
| **46** | 4,8 | 4,2 | 3,0 | 7,9 | 2,0 | 23,3 | 1,2 | 2,3 | 5,5 | 0,9 | 1,2 | 2,1 | 1,7 | 3,0 | 8,2 |
| **47** | 6,4 | 5,1 | 3,8 | 11,1 | 2,5 | 28,4 | 1,4 | 2,7 | 6,1 | 1,3 | 1,3 | 2,5 | 2,4 | 3,4 | 9,8 |
| **48** | 6,9 | 6,0 | 4,3 | 12,1 | 2,7 | 35,5 | 1,5 | 2,9 | 7,0 | 1,3 | 1,4 | 2,9 | 2,6 | 3,9 | 12,0 |
| **49** | 1,8 | 3,0 | 3,0 | 4,4 | 2,4 | 17,0 | 0,8 | 2,3 | 5,4 | 0,7 | 1,2 | 2,1 | 1,5 | 3,2 | 8,1 |
| **50** | 6,3 | 5,1 | 3,6 | 10,2 | 3,0 | 30,2 | 1,4 | 3,0 | 7,0 | 0,8 | 1,5 | 2,4 | 1,8 | 4,0 | 10,2 |
| **51** | 7,4 | 5,4 | 4,2 | 10,8 | 3,3 | 33,2 | 1,4 | 3,2 | 7,4 | 1,3 | 1,7 | 3,2 | 2,7 | 4,4 | 13,4 |
| **52** | 7,4 | 5,5 | 4,1 | 13,4 | 3,3 | 34,9 | 1,6 | 3,3 | 7,6 | 1,8 | 1,8 | 3,4 | 2,6 | 4,1 | 12,3 |
| **53** | 7,7 | 5,4 | 3,9 | 13,2 | 3,2 | 37,5 | 1,5 | 3,3 | 7,8 | 1,6 | 1,8 | 3,4 | 2,4 | 4,0 | 12,0 |
| **54** | 8,5 | 5,8 | 4,2 | 14,8 | 3,5 | 41,8 | 1,6 | 3,4 | 8,3 | 1,7 | 1,9 | 3,4 | 2,6 | 4,2 | 12,7 |
| **55** | 11,5 | 1,6 | 4,0 | 3,7 | 1,3 | 13,5 | 1,0 | 1,8 | 3,7 | 0,5 | 0,8 | 1,6 | 1,5 | 2,4 | 6,7 |
| **56** | 7,2 | 2,4 | 3,7 | 3,5 | 1,6 | 13,2 | 1,2 | 2,0 | 4,2 | 0,4 | 1,0 | 1,9 | 1,7 | 2,5 | 7,3 |
| **57** | 8,2 | 3,4 | 4,4 | 3,8 | 1,7 | 11,6 | 1,5 | 2,5 | 5,5 | 0,4 | 1,1 | 2,2 | 2,3 | 3,2 | 9,2 |
| **58** | 7,5 | 2,3 | 3,6 | 4,1 | 1,6 | 21,0 | 1,2 | 2,2 | 5,0 | 0,7 | 1,2 | 2,4 | 1,7 | 2,8 | 8,0 |
| **59** | 6,3 | 2,9 | 3,6 | 5,1 | 1,8 | 19,4 | 1,4 | 2,4 | 5,3 | 0,7 | 1,2 | 2,6 | 2,1 | 3,2 | 9,5 |
| **60** | 6,7 | 3,6 | 3,9 | 7,1 | 2,0 | 24,7 | 1,6 | 2,7 | 6,1 | 0,7 | 1,3 | 3,0 | 2,5 | 3,5 | 10,2 |
| **61** | 8,2 | 3,1 | 4,2 | 11,3 | 2,6 | 36,5 | 1,7 | 3,1 | 6,8 | 1,3 | 1,6 | 3,7 | 2,6 | 3,7 | 12,0 |
| **62** | 7,0 | 3,8 | 3,9 | 10,7 | 2,4 | 30,1 | 1,7 | 3,0 | 6,9 | 1,2 | 1,5 | 3,3 | 2,6 | 3,8 | 11,7 |
| **63** | 7,2 | 4,6 | 4,0 | 12,2 | 2,6 | 30,9 | 1,9 | 3,4 | 7,5 | 1,2 | 1,6 | 3,5 | 2,9 | 4,2 | 13,4 |
| **64** | 8,9 | 4,2 | 4,2 | 2,6 | 1,4 | 15,0 | 1,1 | 1,9 | 3,4 | 0,5 | 0,9 | 1,7 | 1,3 | 2,2 | 6,1 |
| **65** | 4,6 | 5,6 | 4,9 | 2,3 | 1,5 | 18,1 | 1,3 | 2,3 | 4,1 | 0,5 | 1,0 | 2,0 | 1,8 | 2,8 | 7,8 |
| **66** | 9,2 | 8,0 | 6,2 | 4,2 | 1,7 | 20,1 | 1,5 | 2,7 | 4,7 | 0,5 | 1,2 | 2,3 | 2,1 | 3,4 | 8,8 |
| **67** | 8,8 | 4,5 | 4,2 | 4,6 | 1,8 | 25,2 | 1,7 | 2,9 | 6,5 | 1,5 | 1,5 | 3,7 | 2,7 | 3,7 | 12,1 |
| **68** | 7,5 | 4,9 | 4,0 | 5,8 | 2,0 | 23,6 | 1,7 | 3,0 | 6,5 | 1,3 | 1,4 | 3,1 | 2,6 | 3,4 | 10,7 |
| **69** | 7,0 | 6,0 | 4,3 | 6,2 | 2,2 | 26,1 | 1,9 | 3,3 | 7,2 | 1,5 | 1,6 | 1,7 | 2,8 | 3,8 | 12,0 |
| **70** | 9,1 | 5,1 | 4,5 | 10,6 | 2,4 | 32,4 | 1,7 | 2,9 | 6,5 | 1,5 | 1,5 | 3,7 | 2,7 | 3,7 | 12,1 |
| **71** | 6,8 | 5,2 | 3,9 | 10,9 | 2,3 | 27,4 | 1,7 | 3,0 | 6,5 | 1,3 | 1,4 | 3,1 | 2,6 | 3,4 | 10,7 |
| **72** | 7,0 | 5,6 | 4,0 | 11,0 | 2,4 | 27,8 | 1,9 | 3,3 | 7,2 | 1,5 | 1,7 | 1,7 | 2,8 | 3,8 | 12,0 |
| **73** | 10,4 | 4,7 | 5,3 | 4,3 | 1,5 | 19,6 | 1,2 | 2,2 | 4,6 | 0,5 | 1,0 | 2,2 | 1,5 | 2,6 | 6,1 |
| **74** | 9,7 | 6,1 | 5,7 | 4,9 | 1,7 | 21,0 | 1,5 | 2,6 | 5,3 | 0,6 | 1,1 | 2,4 | 1,9 | 2,8 | 7,2 |
| **75** | 9,0 | 8,1 | 6,6 | 6,1 | 1,9 | 25,3 | 1,7 | 3,0 | 6,3 | 0,4 | 1,3 | 2,4 | 2,5 | 3,3 | 9,5 |
| **76** | 9,0 | 5,2 | 4,8 | 7,0 | 2,1 | 30,0 | 1,5 | 3,0 | 6,7 | 1,0 | 1,5 | 3,3 | 2,4 | 3,8 | 10,8 |
| **77** | 7,1 | 5,5 | 4,4 | 6,4 | 2,2 | 24,0 | 1,6 | 3,0 | 6,3 | 1,0 | 1,4 | 3,2 | 2,5 | 3,7 | 10,6 |
| **78** | 7,5 | 6,3 | 4,7 | 7,7 | 2,4 | 28,0 | 1,9 | 3,3 | 6,9 | 1,0 | 1,6 | 3,2 | 2,9 | 3,9 | 11,7 |
| **79** | 7,0 | 5,4 | 3,9 | 11,0 | 2,5 | 26,5 | 1,9 | 3,3 | 6,9 | 1,4 | 1,5 | 2,8 | 2,8 | 3,9 | 11,4 |
| **80** | 6,5 | 5,5 | 3,8 | 10,8 | 2,5 | 24,2 | 2,0 | 3,5 | 7,2 | 1,6 | 1,7 | 3,2 | 3,2 | 4,0 | 11,9 |
| **81** | 6,7 | 5,9 | 4,1 | 11,9 | 2,7 | 27,6 | 2,1 | 3,7 | 7,7 | 1,3 | 1,5 | 3,0 | 2,9 | 4,0 | 12,0 |

Figure S1. Overlayed mass spectra of oxidized OOL standard under conditions #42 (red) and #49 (black) analysis of oxidized rapeseed oil indicating m/z regions selected for the first stage of selectivity.

Table S4 Summarized ANOVA table for 1ox, 2ox, 3ox OOO, OOL in oxidized standard and oil. Factor A represents the solvent additives, B the solvent additive concentration (mM), C sheath gas temperature (^o^C), D capillary voltage (V) and E nozzle voltage (V).

|  | **1ox OOO** | | | | **2ox OOO** | | | |
| --- | --- | --- | --- | --- | --- | --- | --- | --- |
|  | **Sum of Squares** | **Df** | **F value** | **P value** | **Sum of Squares** | **Df** | **F value** | **P value** |
| A | 0.026028 | 2 | 37.2737 | 7.364e-09 | 0.026028 | 2 | 37.2737 | 7.364e-09 |
| B | 0.007376 | 2 | 10.5624 | 0.0003368 | 0.007376 | 2 | 10.5624 | 0.0003368 |
| C | 0.026701 | 2 | 38.2370 | 5.600e-09 | 0.026701 | 2 | 38.2370 | 5.600e-09 |
| D | 0.006967 | 2 | 9.9774 | 0.0004766 | 0.006967 | 2 | 9.9774 | 0.0004766 |
| E | 0.000264 | 2 | 0.3780 | 0.6884311 | 0.000264 | 2 | 0.3780 | 0.6884311 |
| A × B | 0.019997 | 4 | 14.3184 | 1.199e-06 | 0.019997 | 4 | 14.3184 | 1.199e-06 |
| A × C | 0.021571 | 4 | 15.4453 | 5.764e-07 | 0.021571 | 4 | 15.4453 | 5.764e-07 |
| A x D | 0.007048 | 4 | 5.0464 | 0.0031281 | 0.007048 | 4 | 5.0464 | 0.0031281 |
| A × E | 0.000036 | 4 | 0.0255 | 0.9986614 | 0.000036 | 4 | 0.0255 | 0.9986614 |
| B x C | 0.002933 | 4 | 2.1001 | 0.1055240 | 0.002933 | 4 | 2.1001 | 0.1055240 |
| B x D | 0.000177 | 4 | 0.1268 | 0.9715742 | 0.000177 | 4 | 0.1268 | 0.9715742 |
| B x E | 0.000721 | 4 | 0.5164 | 0.7242166 | 0.000721 | 4 | 0.5164 | 0.7242166 |
| C x D | 0.004082 | 4 | 2.9230 | 0.0373729 | 0.004082 | 4 | 2.9230 | 0.0373729 |
| C x E | 0.000370 | 4 | 0.2651 | 0.8980521 | 0.000370 | 4 | 0.2651 | 0.8980521 |
| D x E | 0.000133 | 4 | 0.0952 | 0.9832118 | 0.000133 | 4 | 0.0952 | 0.9832118 |
|  | **3ox OOO** | | | | **1oxOOL** | | | |
|  | **Sum of Squares** | **Df** | **F value** | **P value** | **Sum of Squares** | **Df** | **F value** | **P value** |
| A | 0.00054108 | 2 | 16.627 | 1.381e-0 | 0.077808 | 2 | 58.6692 | 4.286e-11 |
| B | 0.001075 | 2 | 33.040 | 2.614e-0 | 0.015609 | 2 | 11.7699 | 0.0001685 |
| C | 0.000092 | 2 | 2.839 | 0.0742 | 0.017250 | 2 | 13.0067 | 8.559e-05 |
| D | 0.000065 | 2 | 2.011 | 0.1514 | 0.016486 | 2 | 12.4307 | 0.0001169 |
| E | 0.000008 | 2 | 0.259 | 0.7729 | 0.001452 | 2 | 1.0947 | 0.3476233 |
| A × B | 0.002406 | 4 | 36.971 | 3.420e-1 | 0.067768 | 4 | 25.5494 | 2.746e-09 |
| A × C | 0.000216 | 4 | 3.332 | 0.0226 | 0.049135 | 4 | 18.5245 | 9.173e-08 |
| A x D | 0.000254 | 4 | 3.911 | 0.0113 | 0.028188 | 4 | 10.6272 | 1.745e-05 |
| A × E | 0.000008 | 4 | 0.128 | 0.9707 | 0.002856 | 4 | 1.0767 | 0.3854715 |
| B x C | 0.000283 | 4 | 4.355 | 0.0067 | 0.011450 | 4 | 4.3168 | 0.0070789 |
| B x D | 0.000037 | 4 | 0.574 | 0.6831 | 0.000496 | 4 | 0.1870 | 0.9433422 |
| B x E | 0.000014 | 4 | 0.215 | 0.9275 | 0.002744 | 4 | 1.0344 | 0.4057757 |
| C x D | 0.000040 | 4 | 0.622 | 0.6497 | 0.008208 | 4 | 3.0947 | 0.0302347 |
| C x E | 0.000034 | 4 | 0.530 | 0.7144 | 0.001808 | 4 | 0.6817 | 0.6101276 |
| D x E | 0.000101 | 4 | 1.564 | 0.2093 | 0.002227 | 4 | 0.8398 | 0.5109147 |
|  | **2oxOOL** | | | | **3oxOOL** | | | |
|  | **Sum of Squares** | **Df** | **F value** | **P value** | **Sum of Squares** | **Df** | **F value** | **P value** |
| A | 1.8676 | 2 | 112.1804 | 1.189e-14 | 0.37122 | 2 | 158.8890 | < 2.2e-16 |
| B | 0.3339 | 2 | 20.0537 | 2.954e-06 | 0.07525 | 2 | 32.2106 | 3.395e-08 |
| C | 0.4233 | 2 | 25.4274 | 3.477e-07 | 0.08066 | 2 | 34.5251 | 1.656e-08 |
| D | 0.3275 | 2 | 19.6723 | 3.480e-06 | 0.06582 | 2 | 28.1726 | 1.298e-07 |
| E | 0.0053 | 2 | 0.3203 | 0.7283763 | 0.00029 | 2 | 0.1236 | 0.8841554 |
| A × B | 0.9651 | 4 | 28.9850 | 6.388e-10 | 0.29900 | 4 | 63.9901 | 2.961e-14 |
| A × C | 0.2963 | 4 | 8.8987 | 7.323e-05 | 0.11153 | 4 | 23.8680 | 5.923e-09 |
| A x D | 0.2081 | 4 | 6.2495 | 0.0008805 | 0.03804 | 4 | 8.1419 | 0.0001434 |
| A × E | 0.0078 | 4 | 0.2357 0 | 0.9159971 | 0.00277 | 4 | 0.5925 | 0.6706896 |
| B x C | 0.0960 | 4 | 2.8829 0 | 0.0392836 | 0.01866 | 4 | 3.9945 | 0.0102770 |
| B x D | 0.0048 | 4 | 0.1440 0 | 0.9642352 | 0.00311 | 4 | 0.6660 | 0.6205902 |
| B x E | 0.0391 | 4 | 1.1748 0 | 0.3417061 | 0.00732 | 4 | 1.5667 | 0.2087022 |
| C x D | 0.0583 | 4 | 1.7502 0 | 0.1651102 | 0.01081 | 4 | 2.3142 | 0.0803279 |
| C x E | 0.0161 | 4 | 0.4836 0 | 0.7475769 | 0.00391 | 4 | 0.8373 | 0.5123801 |
| D x E | 0.1402 | 4 | 4.2119 0 | 0.0079858 | 0.00364 | 4 | 0.7781 | 0.5482341 |
|  | **1ox OOO Oil** | | | | **2ox OOO Oil** | | | |
|  | **Sum of Squares** | **Df** | **F value** | **P value** | **Sum of Squares** | **Df** | **F value** | **P value** |
| A | 0.024664 | 2 | 13.9790 | 5.130e-05 | 0.053965 | 2 | 29.8582 | 7.307e-08 |
| B | 0.031192 | 2 | 17.6794 | 8.458e-06 | 0.011757 | 2 | 6.5051 | 0.004500 |
| C | 0.017503 | 2 | 9.9207 | 0.0004931 | 0.073814 | 2 | 40.8408 | 2.736e-09 |
| D | 0.005198 | 2 | 2.9460 | 0.0678953 | 0.013021 | 2 | 7.2043 | 0.002785 |
| E | 0.000022 | 2 | 0.0122 | 0.9878552 | 0.001177 | 2 | 0.6514 | 0.528510 |
| A × B | 0.078591 | 4 | 22.2724 | 1.276e-08 | 0.045193 | 4 | 12.5024 | 4.224e-06 |
| A × C | 0.084257 | 4 | 23.8780 | 5.895e-09 | 0.074104 | 4 | 20.5006 | 3.140e-08 |
| A x D | 0.010742 | 4 | 3.0443 | 0.0321706 | 0.005213 | 4 | 1.4421 | 0.244466 |
| A × E | 0.000533 | 4 | 0.1510 | 0.9610749 | 0.003325 | 4 | 0.9197 | 0.465385 |
| B x C | 0.024063 | 4 | 6.8193 | 0.0004988 | 0.006442 | 4 | 1.7821 | 0.158503 |
| B x D | 0.001947 | 4 | 0.5517 | 0.6992759 | 0.001781 | 4 | 0.4928 | 0.741045 |
| B x E | 0.000733 | 4 | 0.2076 | 0.9321756 | 0.002241 | 4 | 0.6200 | 0.651751 |
| C x D | 0.003658 | 4 | 1.0366 | 0.4046984 | 0.005361 | 4 | 1.4832 | 0.232060 |
| C x E | 0.001901 | 4 | 0.5388 | 0.7083365 | 0.001728 | 4 | 0.4779 | 0.751625 |
| D x E | 0.000676 | 4 | 0.1916 | 0.9408907 | 0.000410 | 4 | 0.1135 | 0.976800 |
|  | **3ox OOO Oil** | | | | **1ox OOL Oil** | | | |
|  | **Sum of Squares** | **Df** | **F value** | **P value** | **Sum of Squares** | **Df** | **F value** | **P value** |
| A | 0.003107 | 2 | 6.7701 | 0.003745 | 0.108347 | 2 | 23.6556 | 6.811e-07 |
| B | 0.006229 | 2 | 13.5706 | 6.347e-05 | 0.042236 | 2 | 9.2214 | 0.0007557 |
| C | 0.006781 | 2 | 14.7723 | 3.421e-05 | 0.031600 | 2 | 6.8993 | 0.0034270 |
| D | 0.001818 | 2 | 3.9607 | 0.029755 | 0.032487 | 2 | 7.0929 | 0.0030032 |
| E | 0.000052 | 2 | 0.1144 | 0.892287 | 0.002597 | 2 | 0.5669 | 0.5732277 |
| A × B | 0.015418 | 4 | 16.7941 | 2.507e-07 | 0.126230 | 4 | 13.7801 | 1.723e-06 |
| A × C | 0.004195 | 4 | 4.5694 | 0.005313 | 0.078841 | 4 | 8.6067 | 9.458e-05 |
| A x D | 0.002125 | 4 | 2.3152 | 0.080224 | 0.035660 | 4 | 3.8929 | 0.0115755 |
| A × E | 0.000362 | 4 | 0.3949 | 0.810626 | 0.001687 | 4 | 0.1841 | 0.9448425 |
| B x C | 0.002065 | 4 | 2.2497 | 0.087193 | 0.023039 | 4 | 2.5151 | 0.0622775 |
| B x D | 0.000350 | 4 | 0.3817 | 0.819916 | 0.001192 | 4 | 0.1302 | 0.9701968 |
| B x E | 0.000394 | 4 | 0.4293 | 0.786288 | 0.006125 | 4 | 0.6686 | 0.6188210 |
| C x D | 0.001055 | 4 | 1.1501 | 0.352307 | 0.021027 | 4 | 2.2954 | 0.0822684 |
| C x E | 0.000113 | 4 | 0.1237 | 0.972825 | 0.000555 | 4 | 0.0606 | 0.9928464 |
| D x E | 0.000568 | 4 | 0.6191 | 0.652325 | 0.013508 | 4 | 1.4746 | 0.2346008 |
|  | **2ox OOL Oil** | | | | **3ox OOL Oil** | | | |
|  | **Sum of Squares** | **Df** | **F value** | **P value** | **Sum of Squares** | **Df** | **F value** | **P value** |
| A | 0.40993 | 2 | 43.3918 | 1.400e-09 | 0.075946 | 2 | 29.4548 | 8.368e-08 |
| B | 0.08319 | 2 | 8.8059 | 0.0009797 | 0.046075 | 2 | 17.8697 | 7.752e-06 |
| C | 0.33567 | 2 | 35.5315 | 1.224e-08 | 0.044576 | 2 | 17.2883 | 1.013e-05 |
| D | 0.08504 | 2 | 9.0016 | 0.0008665 | 0.021325 | 2 | 8.2708 | 0.001378 |
| E | 0.00206 | 2 | 0.2178 | 0.8055445 | 0.002664 | 2 | 1.0331 | 0.368224 |
| A × B | 0.24418 | 4 | 12.9233 | 3.125e-06 | 0.106256 | 4 | 20.6049 | 2.974e-08 |
| A × C | 0.22404 | 4 | 11.8575 | 6.781e-06 | 0.046134 | 4 | 8.9462 | 7.028e-05 |
| A x D | 0.05937 | 4 | 3.1423 | 0.0285169 | 0.012250 | 4 | 2.3755 | 0.074311 |
| A × E | 0.00506 | 4 | 0.2681 | 0.8961788 | 0.002347 | 4 | 0.4551 | 0.767876 |
| B x C | 0.06145 | 4 | 3.2522 | 0.0249304 | 0.015707 | 4 | 3.0458 | 0.032108 |
| B x D | 0.00328 | 4 | 0.1734 | 0.9503060 | 0.000771 | 4 | 0.1494 | 0.961811 |
| B x E | 0.01696 | 4 | 0.8977 | 0.4775906 | 0.005118 | 4 | 0.9924 | 0.426798 |
| C x D | 0.04034 | 4 | 2.1349 | 0.1009353 | 0.007931 | 4 | 1.5381 | 0.216444 |
| C x E | 0.00454 | 4 | 0.2403 | 0.9132620 | 0.000576 | 4 | 0.1117 | 0.977443 |
| D x E | 0.01542 | 4 | 0.8162 | 0.5249727 | 0.006924 | 4 | 1.3426 | 0.277174 |


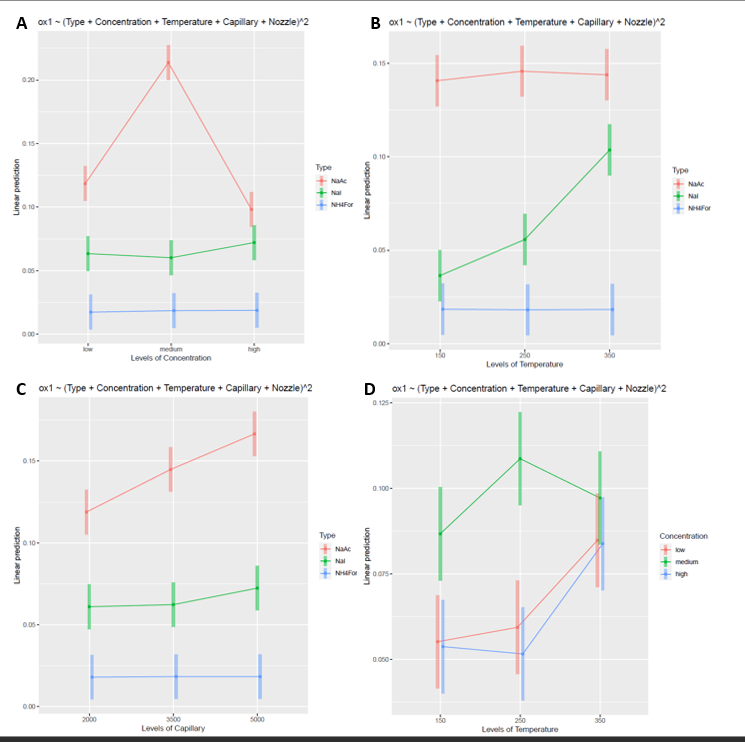


Figure S2 Two-way interactions for 1oxOOO A. Type x Concentration B. Type x Temperature C. Type x Capillary D. Concentration x Temperature.


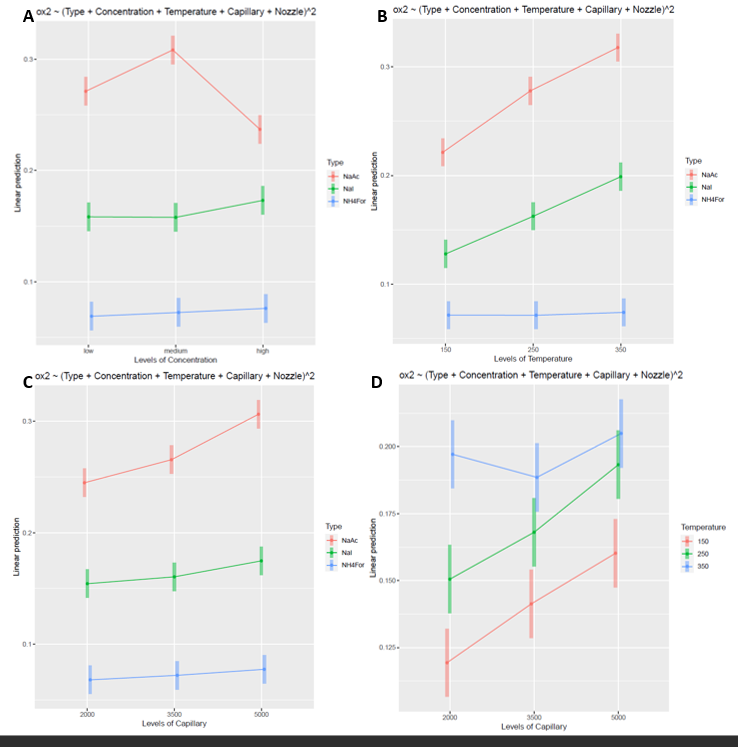


Figure S3 Two-way interactions for 2ox-OOO A. Type x Concentration B. Type x Temperature C. Type x Capillary D. Temperature x Capillary.


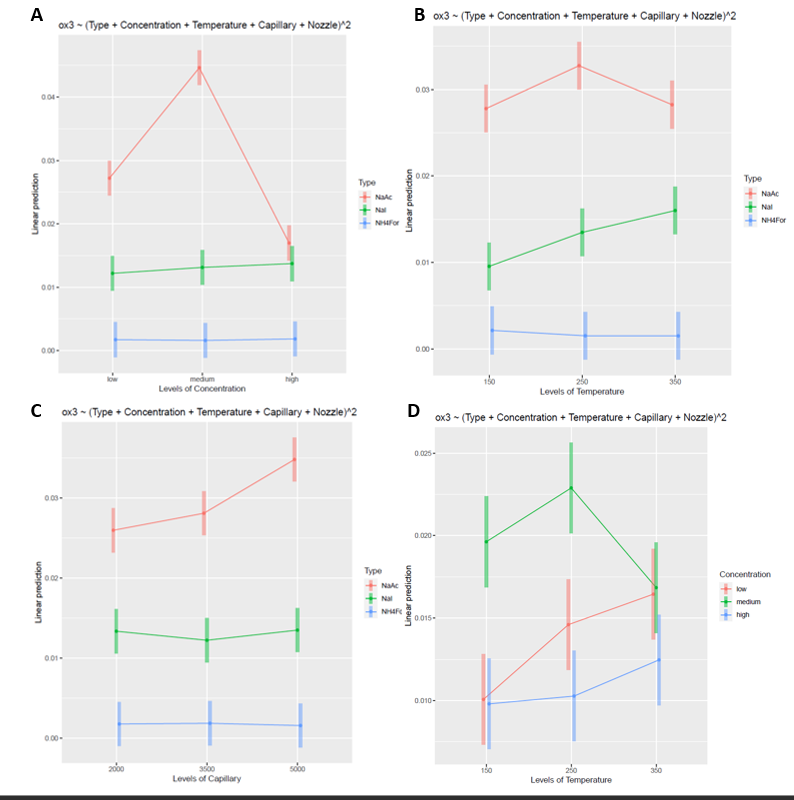


Figure S4 Two-way interactions for 3ox-OOO A. Type x Concentration B. Type x Temperature C. Type x Capillary D. Concentration x Temperature.


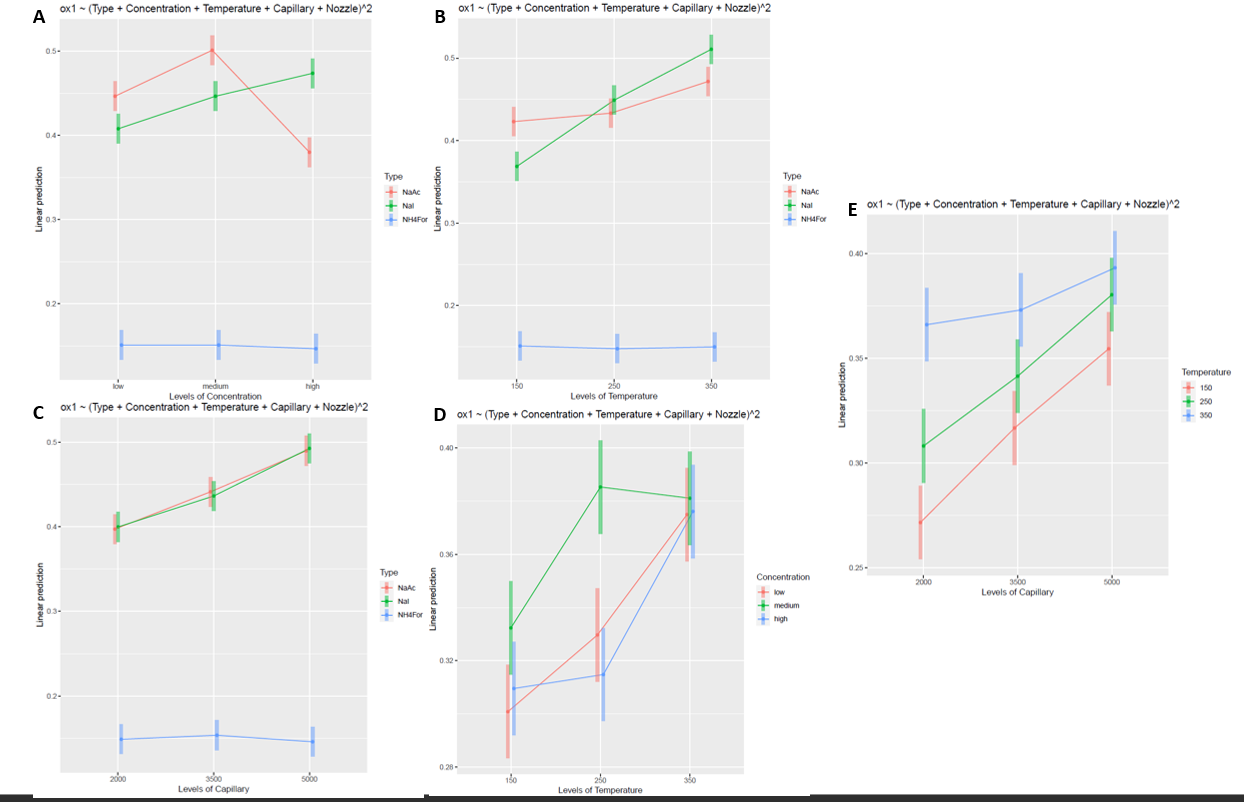


Figure S5 Two-way interactions for 1ox-OOL A. Type x Concentration B. Type x Temperature C. Type x Capillary D. Concentration x Temperature E. Temperature x Capillary.


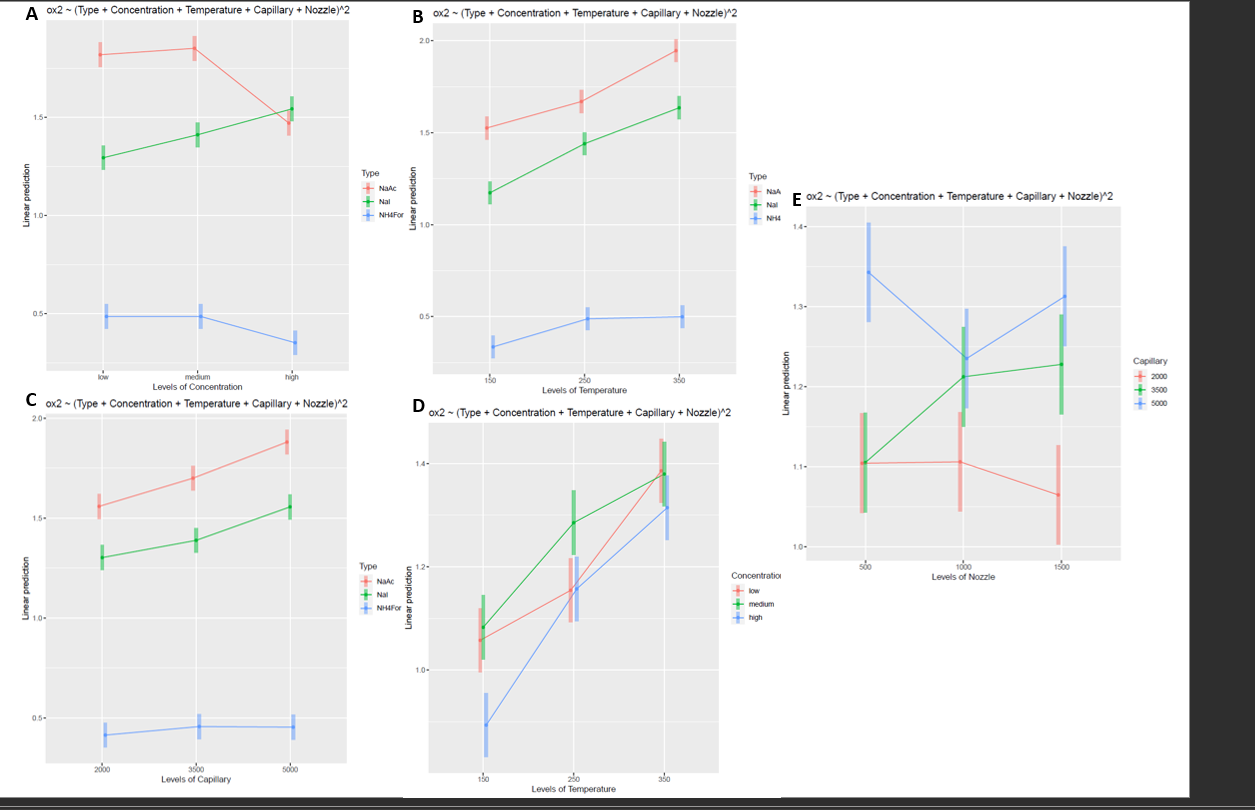


Figure S6 Two-way interactions for 2ox-OOL A. Type x Concentration B. Type x Temperature C. Type x Capillary D. Concentration x Temperature E. Temperature x Capillary.


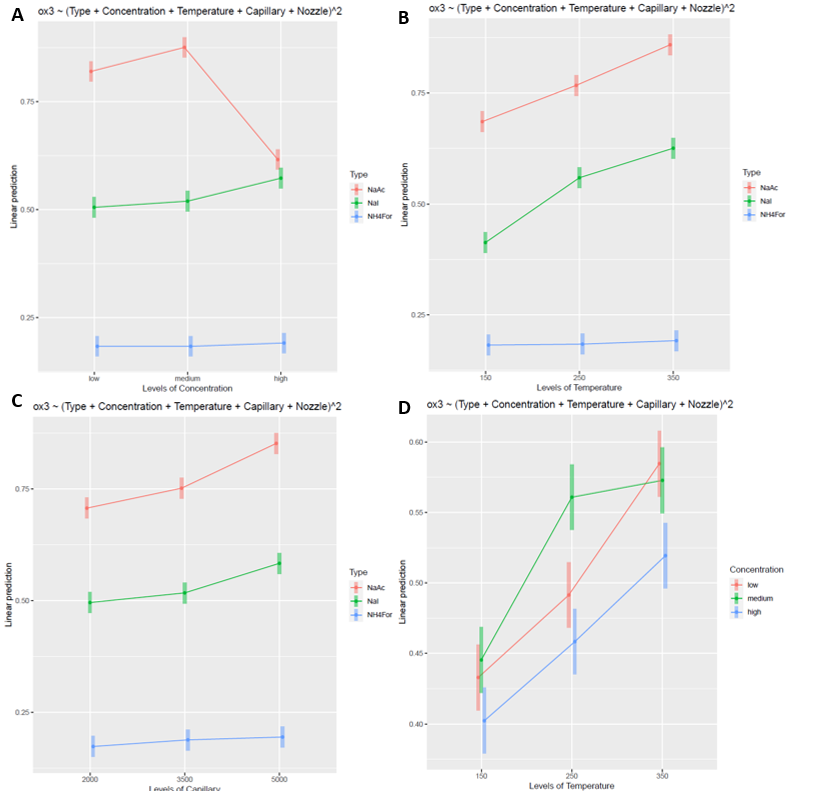


Figure S7 Two-way interactions for 3ox-OOL A. Type x Concentration B. Type x Temperature C. Type x Capillary D. Concentration x Temperature.


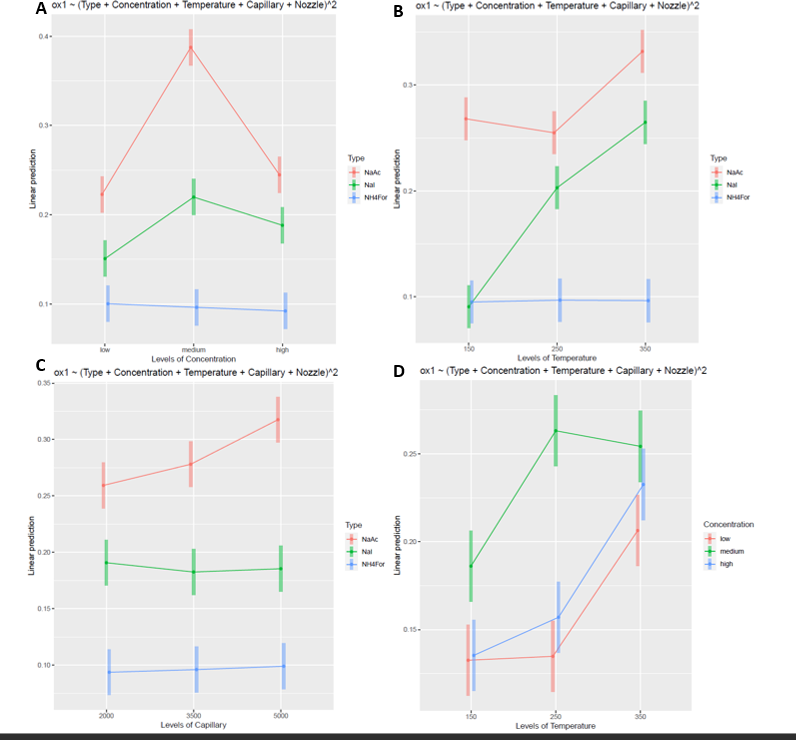


Figure S8 Two-way interactions for 1ox-OOO Oil A. Type x Concentration B. Type x Temperature C. Type x Capillary D. Concentration x Temperature.


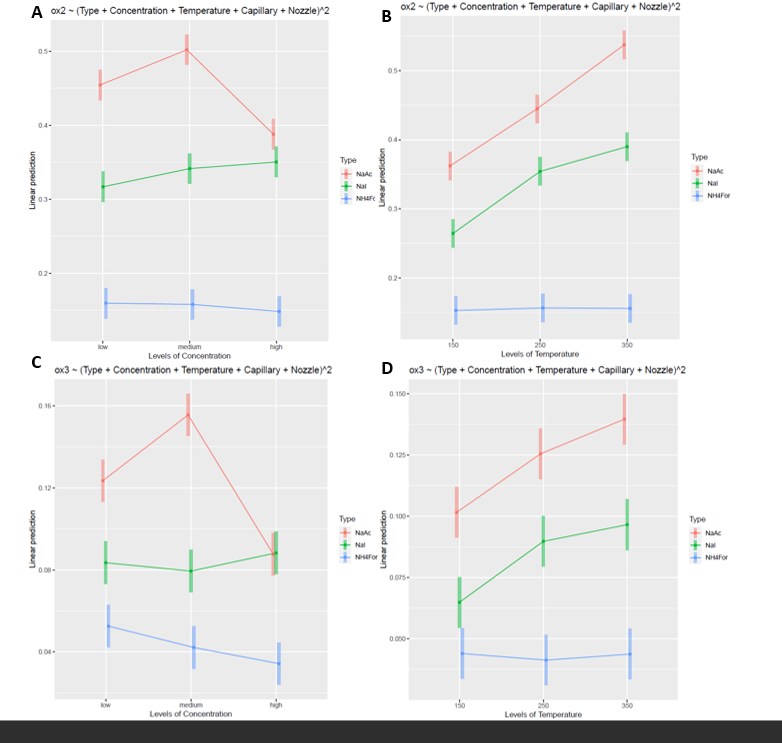


Figure S9 Two-way interactions for 2 & 3 ox-OOO Oil A. 2 ox Type x Concentration B. 2ox Type x Temperature C. 3ox Type x Concentration D. 3ox Type x Temperature.


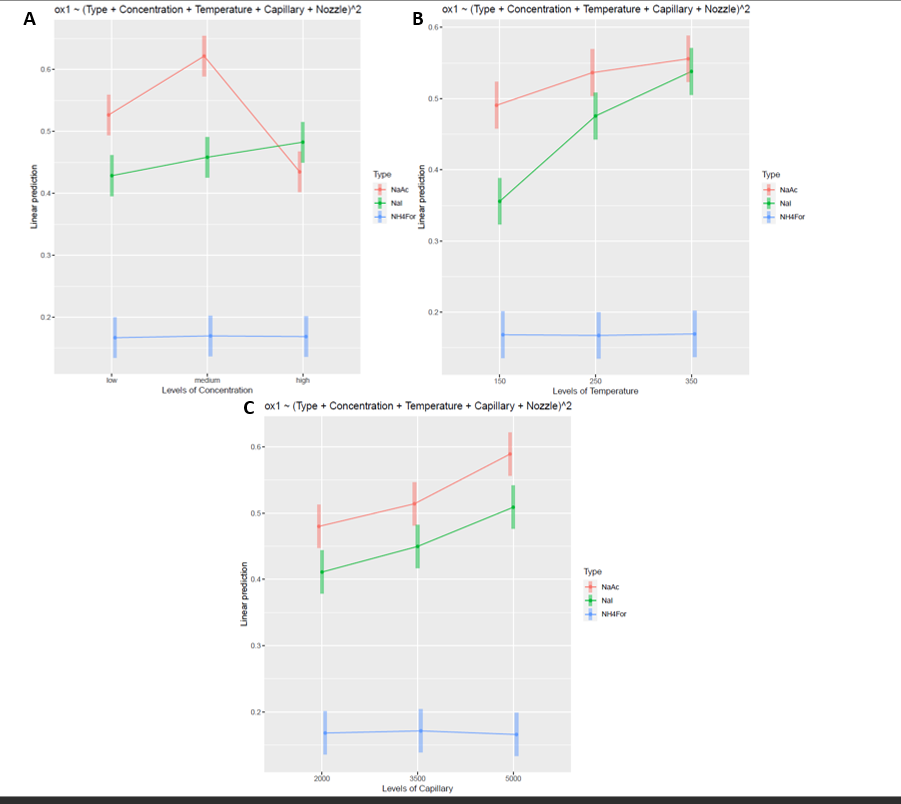


Figure S10 Two-way interactions for 1ox-OOL Oil A. Type x Concentration B. Type x Temperature C. Type x Capillary.


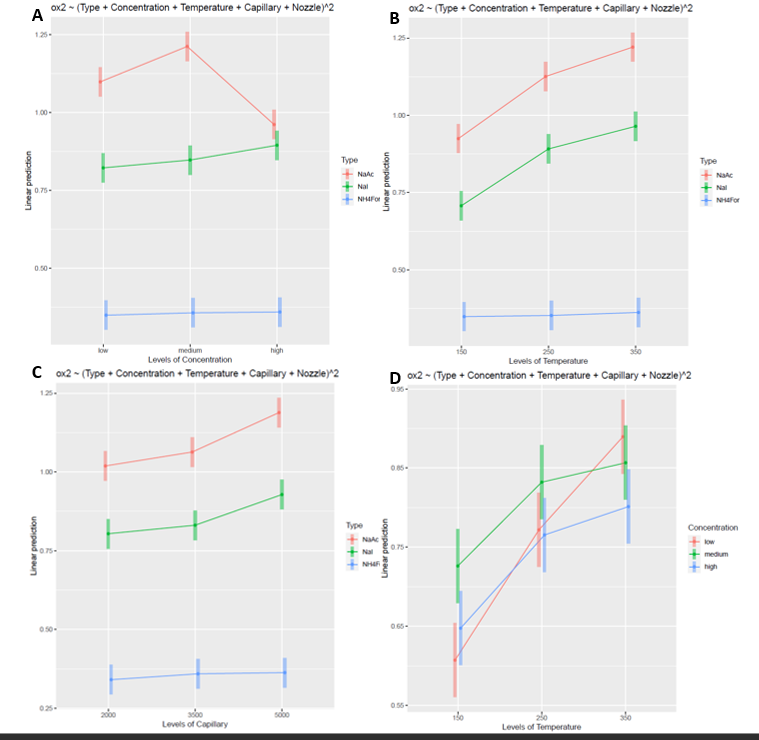


Figure S11 Two-way interactions for 2ox-OOL Oil A. Type x Concentration B. Type x Temperature C. Type x Capillary D. Concentration x Temperature.


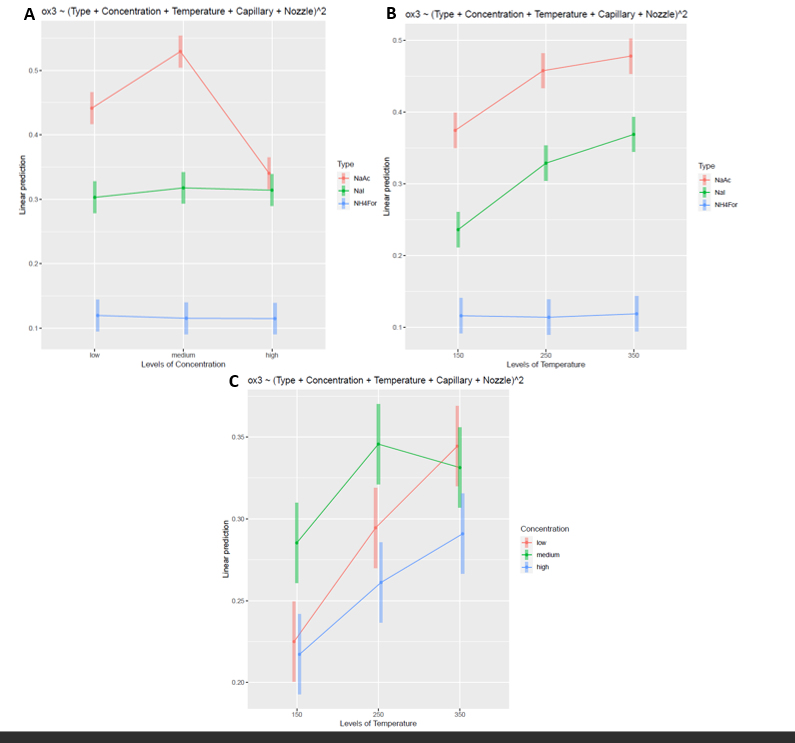


Figure S12 Two-way interactions for 3ox-OOL Oil A. Type x Concentration B. Type x Temperature C. Concentration x Temperature.

Table S5 Validation results of combination of factors indicated by statistical analysis. Factor A represents the solvent additives, B the solvent additive concentration (mM), C sheath gas temperature (^o^C), D capillary voltage (V) and E nozzle voltage (V).

| **Factors** | | | | |
| --- | --- | --- | --- | --- |
| **A** | **B** | **C** | **D** | **E** |
| NaOAc | 0,1 | 250 | 5000 | 1000 |
|  |  | **1ox** | **2ox** | **3ox** |
| **Standard** | **OOO** | 11,2±0,4 | 3,2±0,4 | 26,7±2,6 |
|  | **OOL** | 1,5±0,1 | 3,2±0,3 | 7,4±1 |
| **Oil** | **OOO** | 1,5±0,1 | 1,7±0,2 | 3,4±0,4 |
|  | **OOL** | 2,6±0,1 | 4,0±0,4 | 12,1±1,8 |
